# Podocalyxin-Like Protein Is Expressed in Glioblastoma Multiforme Stem-Like Cells and Is Associated with Poor Outcome

**DOI:** 10.1371/journal.pone.0075945

**Published:** 2013-10-16

**Authors:** Zev A. Binder, I-Mei Siu, Charles G. Eberhart, Colette ap Rhys, Ren-Yuan Bai, Verena Staedtke, Hao Zhang, Nicolas R. Smoll, Steven Piantadosi, Sara G. Piccirillo, Francesco DiMeco, Jon D. Weingart, Angelo Vescovi, Alessandro Olivi, Gregory J. Riggins, Gary L. Gallia

**Affiliations:** 1 Department of Neurosurgery, Johns Hopkins University School of Medicine, Baltimore, Maryland, United States of America; 2 Johns Hopkins Physical Science Oncology Center and Institute for NanoBioTechnology, Johns Hopkins University, Baltimore, Maryland, United States of America; 3 Department of Pathology, Johns Hopkins University School of Medicine, Baltimore, Maryland, United States of America; 4 Department of Neurology, Johns Hopkins University School of Medicine, Baltimore, Maryland, United States of America; 5 Department of Molecular Microbiology and Immunology, Johns Hopkins University School of Public Health, Baltimore, Maryland, United States of America; 6 Gippsland Medical School, Monash University, Churchill, Victoria, Australia; 7 Department of Oncology Biostatistics, Johns Hopkins University School of Medicine, Baltimore, Maryland, United States of America; 8 StemGen, Milan, Italy; 9 Department of Neurosurgery, Istituto Nazionale Neurologico C. Besta, Milan, Italy; 10 Department of Oncology, Johns Hopkins University School of Medicine, Baltimore, Maryland, United States of America; 11 Department of Biotechnology and Biosciences, University of Milano Biocca, Milan, Italy; University of Florida, United States of America

## Abstract

Glioblastoma multiforme (GBM) is the most common primary malignant adult brain tumor and is associated with poor survival. Recently, stem-like cell populations have been identified in numerous malignancies including GBM. To identify genes whose expression is changed with differentiation, we compared transcript profiles from a GBM oncosphere line before and after differentiation. Bioinformatic analysis of the gene expression profiles identified podocalyxin-like protein (PODXL), a protein highly expressed in human embryonic stem cells, as a potential marker of undifferentiated GBM stem-like cells. The loss of PODXL expression upon differentiation of GBM stem-like cell lines was confirmed by quantitative real-time PCR and flow cytometry. Analytical flow cytometry of numerous GBM oncosphere lines demonstrated PODXL expression in all lines examined. Knockdown studies and flow cytometric cell sorting experiments demonstrated that PODXL is involved in GBM stem-like cell proliferation and oncosphere formation. Compared to PODXL-negative cells, PODXL-positive cells had increased expression of the progenitor/stem cell markers Musashi1, SOX2, and BMI1. Finally, PODXL expression directly correlated with increasing glioma grade and was a marker for poor outcome in patients with GBM. In summary, we have demonstrated that PODXL is expressed in GBM stem-like cells and is involved in cell proliferation and oncosphere formation. Moreover, high PODXL expression correlates with increasing glioma grade and decreased overall survival in patients with GBM.

## Introduction

Glioblastoma multiforme (GBM), World Health Organization (WHO) grade IV astrocytoma, is the most common primary malignant adult brain tumor and is treated with a combination of surgery, radiation, and chemotherapy. These tumors remain incurable with a current median survival of 14.6 months [1]. Stem-like cell populations have been identified in a number of malignancies including GBM [2], [3]. GBM stem-like cells are heterogeneous populations that, like normal neural stem cells, are self-renewing and multi-potent. These cells can differentiate along both neuronal and glial lineages [2]. They grow as oncospheres *in vitro* and, when implanted intracranially, form tumors histologically identifiable as GBM [2]. Additionally, there is evidence these stem-like cells are resistant to chemotherapy and radiotherapy [4], [5].

Several methods have been proposed to isolate GBM stem-like cells. One is the use of candidate stem cell markers, such as CD133, CD15, CD44, integrin α_6_, and L1CAM, to isolate the putative stem cell fraction from human GBMs [3], [6]–[9]. There is, however, a lack of consensus regarding these markers in the literature. For example, isolation of the CD133-positive fraction has been shown to miss cells with stem-like functions and several studies have demonstrated that CD133-negative cells exhibit stem cell capabilities [10]–[12]. Similarly, CD15 has literature both supporting the claim of it being a GBM stem-like cell marker [6] and refuting that claim [13]. Although CD44 has been shown to identify cancer stem cells in other pathologies [14], there is controversy about this association with GBM stem-like cells [7], [15]. The data on integrin α_6_ and L1CAM comes from populations first identified by expression of CD133 [8], [9]. These conflicting studies reveal the difficulties involved in using stem cell markers.

Another method to identify these cells is based on the “side population” of cells expressing ATP-binding cassette transporters, which pump out Hoechst 33342 dye [16]. Other studies, however, suggest a lack of specificity with this approach by demonstrating toxicity of Hoechst dye which may have selected cells for their resistance to this compound and not for their stem cell capabilities *per se* [12]. An additional study demonstrated that side population cells were unstable and could arise from non-side population cells [17].

An alternate method to isolate GBM stem-like cells is to use specific culturing conditions, including lack of serum and addition of EGF and FGF-2 [2]. Lee et al. compared the genotypic and phenotypic characteristics of such oncospheres to parental tumors and related adherent GBM cell lines cultured in the presence of serum and absence of EGF and FGF-2, derived from the same parental tumors [18]. This study demonstrated a high degree of similarity among the parental tumors, normal neural stem cell lines, and cells cultured in EGF and FGF-2. The genotypes and phenotypes of the adherent cells diverged significantly from the parental tumors [18].

To identify genes whose expression is changed with oncosphere differentiation, we compared transcript profiles of an undifferentiated GBM stem-like cell line and its differentiated counterpart. One of the genes significantly over-expressed in undifferentiated stem-like cells was podocalyxin-like protein (PODXL). In this study, we functionally characterize this protein in GBM stem-like cells and investigate the clinical significance of expression of PODXL in gliomas.

## Materials and Methods

### Cell lines

The GBM oncosphere lines 020913 (HSR-GBM1), HSR-GBM2, HSR-GBM3, and 060919 have been previously described [2], [19], [20]. The GBM oncosphere line TSC1228 was obtained from Howard Fine at the National Institutes of Health and has also been previously described [18]. Additional malignant glioma oncosphere lines were established from fresh tumor tissue immediately following resection at Johns Hopkins Hospital (JHH), obtained under Johns Hopkins Medicine Office of Human Subjects Research Institutional Review Board approval with informed written consent. The cell lines were authenticated using short tandem repeat profiling (data not shown). All lines were maintained in NeuroCult medium (StemCell Technology, Vancouver, BC, Canada) supplemented with 0.2% heparin (StemCell Technology), hEGF (20 ng/mL, Peprotech, Rocky Hill, NJ), and hFGF-2 (10 ng/mL, Peprotech). Differentiation was performed as previously described [2].

### RNA preparation

Total RNA was prepared with the RNAgents kit (Promega, Madison, WI) following the manufacturer's instructions. RNA quality was assessed by denaturing agarose gel electrophoresis.

### Serial analysis of gene expression (SAGE)

Micro long-SAGE was performed as previously described [21] using 5–10 µg of total RNA isolated from 020913 cells that had either remained undifferentiated or were fully differentiated (incubated in differentiation medium for 28 days) [2]. The tags from these SAGE libraries were then extracted using SAGE2000 v 4.5 software. The full sets of tag counts for these libraries are available online [22], [23].

### Quantitative real-time PCR (qRT-PCR)

cDNAs from matched sets of undifferentiated and differentiated GBM oncosphere lines were generated from total RNA using Superscript II reverse transcriptase according to the manufacturer's instructions (Invitrogen, Carlsbad, CA). qRT-PCR was optimized and performed on a Bio-Rad iCycler machine (Bio-Rad Laboratories, Hercules, CA) as previously described [24] (primer sequences available upon request). At least two independent qRT-PCR reactions were performed in triplicate. Expression was normalized to COX6B, which was shown by SAGE to be at roughly equivalent levels in both SAGE libraries, using iCycler software version 3.0 (Bio-Rad Laboratories). For qRT-PCR of Musashi1, SOX2, and BMI1, RNA was isolated from using Trizol according to the manufacturer's instructions (Invitrogen). cDNA was synthesized using a QuantiTect Reverse Transcription kit according to the manufacturer's instructions (Qiagen, Valencia, CA). qRT-PCR was performed as described above with minor modifications. Expression was normalized to GAPDH (primer sequences available upon request). At least two independent qRT-PCR reactions were performed in quintuplicate. All qRT-PCR products were resolved by electrophoresis on a 3% low-molecular weight agarose/ethidium bromide gel to ensure a single product was amplified.

### Analytical flow cytometry

Oncospheres were triturated to obtain a single-cell suspension, incubated with human and mouse pre-immune IgG (Sigma-Aldrich, St. Louis, MO) for 15 minutes at room temperature, stained with a phycoerythrin-conjugated mouse monoclonal anti-PODXL (R&D Systems, Minneapolis, MN), a phycoerythrin-conjugated mouse monoclonal anti-CD133/2 (Clone 293C, Miltenyi Biotec, Auburn, CA) or a phycoerythrin-conjugated isotype control mouse IgG_2B_ or IgG_2A_ antibody (Miltenyi Biotec, R&D Systems; BD Biosciences, San Diego, CA) for 30 minutes on ice, incubated with 7-amino-actinomycin D (7-AAD) (BD Biosciences), and analyzed on a FACSCalibur (BD Biosciences). Analytical flow cytometry was performed at least twice for each oncosphere line. The percent positivity was determined by using a gate set by 1.0% positive events from the isotype population.

### Viral knockdown of PODXL

A Mission™ TRC-Hs (Human) clone set of five different sequence-verified shRNA lentiviral plasmid vectors targeting PODXL (TRCN0000117017 thru 21) was obtained from the High Throughput Biology Center (Johns Hopkins University, Baltimore, MD). A second set of five different sequence-verified inducible shRNA lentiviral plasmid vectors (pTRIPZ) targeting PODXL (V3THS_385278, V3THS_385276, V2THS_36329, V2THS_36258, and V2THS_36259) was obtained to provide a second shRNA construct (Thermo Scientific, Rockford, IL). The lentiviruses were made as previously described [25]. Cells were cultured for 5 to 7 days and then 10 µL of concentrated virus and 8 ng/mL of Polybrene (Sigma-Aldrich) were added. The medium was changed after 24 hours. For the Mission™ TRC-Hs plasmids, 0.5 µg/mL of puromycin (Sigma-Aldrich) were added to the cultures three days after transduction. For the pTRIPZ plasmids, 2 µg/mL doxycycline (Santa Cruz Biotechnology, Dallas, TX) were added every 48 hours for plasmid induction, both in the knockdown vector and the empty control vector. Change in PODXL expression was determined by flow cytometry, as described above, with minor modifications. For experiments using pTRIPZ plasmids, an Alexa Fluor 488-conjugated mouse monoclonal anti-PODXL antibody and an Alexa Fluor 488-conjugated isotype control mouse IgG_2A_ antibody (R&D Systems) were utilized given the turboRFP (tRFP) expression associated with induction of the plasmids. TRCN0000117018 and V2THS_36258 shRNA sequences demonstrated the greatest reduction of PODXL expression and were selected for further knockdown studies.

### Fluorescence-activated cell sorting (FACS)

The analysis and sorting were performed using Summit 4.3 software (DAKO USA, Carpinteria, CA) on a MoFlo MLS (Beckman Coulter, Brea, CA) sorter equipped with a Coherent Enterprise II 621 laser and a CyCLONE automated cloner. Two primary GBM oncosphere lines, 020913 and JHU-0879, were incubated with anti-PODXL antibody as described above. Gates for the PODXL-positive and PODXL-negative cells were set using an unstained population and an isotype-stained population. The final gates were refined to the top and bottom 2.5–5% of cells based on PODXL expression. In addition, the sorter was utilized to count and plate cells for the proliferation and oncosphere formation assays involving the lentiviral constructs. In the case of the Mission™ TRC-Hs vectors, 7-AAD was utilized to gate on live cells. For the pTRIPZ plasmids, tRFP expression was used to gate on positively-transduced cells.

### Cell proliferation assay

Proliferation was assessed using Alamar Blue (Invitrogen) following the manufacturer's instructions. Cells were counted and plated at a concentration of 500 or 750 cells per 180 µL of complete NeuroCult medium using the MoFlo MLS sorter as described above, with 5 replicates at each concentration, in a 96-well plate. The plates were read daily on a Victor^3^ automated plate reader (Perkin Elmer, Waltham, MA). For lentiviral knockdown and cell-sorting experiments, background fluorescence, determined by Alamar Blue in media alone, was subtracted from the readings. For the pTRIPZ knockdown experiments, background fluorescence was determined by adding 2 µg/mL doxycycline every 48 hours to wells containing media and Alamar Blue. For the Mission™ TRC-Hs plasmids, three independent transductions were performed. For the pTRIPZ plasmids, the proliferation assay was performed in triplicate with independently induced populations. For the PODXL-positive and PODXL-negative sorted populations, two separate cell lines, 020913 and JHU-0879, were used for three separate assays each.

### Oncosphere formation/limiting dilution assay

Cell populations were obtained from lentiviral knockdown and FACS. PODXL-positive or PODXL-negative cells were plated in 80 µL of complete NeuroCult medium in 96-well plates. PODXL-positive cells were plated at concentrations of 1, 2, 5, 10, and 50 cells per well. PODXL-negative cells were plated at concentrations of 1, 10, 50, 200, and 500 cells per well. For each concentration of cells there were 48 wells. Plates were analyzed by light microscopy for oncospheres, 12 to 20 days after plating. Positive wells were defined as groups of cells larger than 125 µm in diameter. Individual spheres from positive wells were triturated, re-plated, and monitored for growth of new oncospheres. For the Mission™ TRC-Hs plasmids, three independent transductions were performed. For the pTRIPZ plasmids, the assay was performed in triplicate with independently induced populations. For the PODXL-sorted populations, the assay was run three times each using two separate cell lines, 020913 and JHU-0879.

### Immunohistochemistry

Formalin-fixed, paraffin-embedded sections of GBMs (8 total tumors) and tissue microarrays (TMAs) of WHO grades I–IV astrocytomas with normal controls were stained as previously described [26] using an anti-PODXL antibody (1∶200, R&D Systems) overnight at 4°C followed by incubation with a goat anti-mouse IgG conjugated to horseradish peroxidase (Dako USA). A total of 148 cases were represented in the TMAs, including 6 WHO grade I astrocytomas (pilocytic astrocytoma), 47 WHO grade II diffuse astrocytomas, 41 WHO grade III anaplastic astrocytomas, and 54 WHO grade IV GBMs. Three commercial TMAs were used (US Biomax, Rockville, MD) and a fourth TMA was generated at JHH (CGE). Each sample on the TMAs was represented by one to three cores and staining intensity was averaged across the replicate cores. Human kidney tissue served as a positive control on the JHH TMA. Intensity of staining was graded as negative, weak, or strong by a single neuropathologist (CGE).

### Tumor sample processing

Primary tumor samples were obtained and processed within 24 hours of resection. A total of 64 samples were processed: 2 grade I astrocytomas, 9 grade II diffuse astrocytomas, 10 grade III anaplastic astrocytomas, and 43 grade IV GBMs. Samples were mechanically dissociated using the two-scalpel method, a tissue douncer, and passaging through a 16-gauge needle. The mixture was then incubated with 10 mg/mL Collagenase IV (Invitrogen) for 15 minutes at 37°C. After the mixture was passed through a 75 µm filter, the cells were incubated with a red blood cell lysis kit (BD Biosciences) for 15 minutes at room temperature and washed in filtered PBS +1% FBS. The resulting pellet was centrifuged in a 30% sucrose solution (w/v) at 3,000 g for 20 minutes. The cells were resuspended in PBS and PODXL expression was analyzed using analytical flow cytometry as described above.

### Caspase assay

Apoptosis was assayed using the Caspase-Glo 3/7 Assay (Promega) on JHU-0879 cells with Mission™ TRC-Hs knockdown of PODXL following the manufacturer's instructions, incubating with Caspase-Glo for 1 hour at room temperature prior to analysis. Each well contained 10,000 cells. As a positive control, cells were treated with 20 µM of the multi-targeted receptor tyrosine kinase inhibitor sunitinib (LC Laboratories, Woburn, MA) at room temperature for three hours.

### Survival Analysis

Kaplan-Meier Survival graphs and statistics were generated on the Repository of Molecular Brain Neoplasia DaTa (REMBRANDT) Database website, a joint initiative of the Neuro-Oncology Branch of the National Cancer Institute and the National Institute of Neurological Disorders and Stroke [23], [27]. In this database, PODXL expression was ascertained using Affymetrix GeneChips®. Up-regulated expression was defined as a ≥2× increase in expression compared to non-tumor brain tissue. Intermediate expression was defined as between a <2× increase and <2× decrease in expression compared to non-tumor samples. Down-regulated expression was defined as a ≥2× decrease in expression compared to non-tumor samples. The only sample in which PODXL expression was down-regulated was excluded from the final analysis. A total of 342 gliomas were analyzed, including 181 GBMs.

### Statistical methods

The statistical significance of differentially expressed genes in the SAGE libraries was assessed using Monte Carlo simulation. RT-PCR data was analyzed using the 2^−ΔΔ*Ct*^ method as previously described [28]. P-values were calculated using a two-sided, paired t-test of the absolute expression values. For analytical flow cytometry of fresh tumor samples, results of PODXL expression were analyzed using a Rank-sum test with a p-value of <0.05 (Stata version 11.1, College Station, Texas). For cell proliferation assays, the linear portion of the resulting growth curve was fit with a linear regression model and a two-sided t-test was performed on the linear regression model (GraphPad, La Jolla, CA). The limiting dilution assay was analyzed as described previously [29] with statistical analysis done using Extreme Limiting Dilution Analysis [30]. Immunohistochemistry results were assessed by cross-tabulating PODXL positivity and tumor grade using chi-square analysis (SAS, Cary, NC). The REMBRANDT survival data were analyzed using a Kaplan-Meier Survival graph and the effect of expression was modeled using Cox-proportional hazard regression (Stata version 11.1).

## Results

### Gene expression profiling of undifferentiated and differentiated GBM stem cells

To compare gene expression profiles of undifferentiated and differentiated GBM stem-like cells, SAGE was performed on undifferentiated and differentiated 020913 cells. Bioinformatic analysis of the gene expression profiles of the undifferentiated and differentiated cells identified numerous genes significantly over-expressed in GBM oncospheres (Table 1, p<0.05); the genes over-expressed in the differentiated GBM oncospheres are shown in Table S1. *PROMININ*, the gene encoding the putative stem cell marker CD133 [3], was modestly decreased from 16 to 12 tags upon differentiation (data not shown), but was not statistically significant at this level of tag sampling. Additionally, expected increases in tag counts were observed for Tuj1 and GFAP upon differentiation (data not shown), but did not reach statistical significance. Most notably, PODXL, a gene expressed in human embryonic stem cells (hESCs) [31]–[37], was highly over-expressed in undifferentiated oncospheres. Further *in silico* analysis of PODXL demonstrated high expression in both hESC and human embryonal carcinoma cell lines (Figure S1).

**Table 1:** Table of genes up-regulated greater than 8 fold in undifferentiated GBM oncospheres.

SAGE tag sequence	UniGene ID	Gene symbol (name)	Fold incr doublets
GGTACCCATT	Hs.298654	DUSP6 (dual specificity phosphatase 6)	19.39
GATTTGCCCT	Hs.652175	TRIB2 (tribbles homolog 2)	16.33
TTTTATAATT	Hs.26770	FABP7 (fatty acid binding protein 7)	13.27
TATATTGTAC	Hs.18676	SPRY2 (sprouty homolog 2)	12.25
AATGACTGAA	Hs.93659	PDIA4 (protein disulfide isomerase family A, member 4)	11.23
GACGCCCTGC	Hs.56663	OLIG1 (oligodendrocyte transcription factor 1)	11.22
GACCCTGGGG	Hs.467151	JOSD2 (josephin domain containing 2)	11.22
CTCAGCAAAC	Hs.404056	EIF3S1 (eukaryotic translation initiation factor 3, subunit 1 alpha, 35 kDa)	10.2
TGGAAGGACC	Hs.5086	MGC10433	10.2
GAGTTTGTCC	Hs.438877	NLGN3 (neuroligin 3)	10.2
GTCAGAACTT	Hs.602085	PHLDA1 (pleckstrin homology-like domain, family A, member 1)	9.18
TCAAATGCAA	Hs.584842	SART3 (squamous cell carcinoma antigen recognized by T cells 3)	9.18
CTACCCTTTC	Hs.363137	TCP1 (t-complex 1)	9.18
ATGCTGCCAA	Hs.304792	PROSC (proline synthetase co-transcribed homolog [bacterial])	9.18
**GAGGACACAG**	**Hs.16426**	**PODXL (podocalyxin-like)**	**8.16**
ATAATTGACT	Hs.15591	COPS6 (COP9 constitutive photomorphogenic homolog subunit 6 [Arabidopsis])	8.16
GAATTACAGT	Hs.653288	RNF12 (ring finger protein 12)	8.16
GAACCTTGAG	Hs.591140	FOXK2 (forkhead box K2)	8.16
GTCTGAGCTC	Hs.66915	C22orf16	8.16
GCCAGACACC	Hs.525232	LRP10 (low density lipoprotein receptor-related protein 10)	8.16
TTCACTGCCG	Hs.78089	ATP6V1F (ATPase, H+ transporting, lysosomal 14 kDa, V1 subunit F)	8.16
TACCTTCCTT	Hs.592164	POFUT2 (protein O-fucosyltransferase 2)	8.16
AGCTATTCCT AAGAACATTG	Hs.368404 Hs.387567	EXT2 (exostoses [multiple] 2) ACLY (ATP citrate lyase)	8.16 8.16
CAAGAAAGCA	Hs.422181	S100B (S100 calcium binding protein B)	8.16
CGCTTTGCGC	Hs.529782	VCP (valosin-containing protein)	8.16
TGTCCCCTCA	Hs.525622	AKT1 (v-Akt murine thymoma viral oncogene homolog 1)	8.16
GTACTAGTGT	Hs.303649	CCL2 (chemokine [C-C motif] ligand 2)	8.16
ATACTGCTGC	Hs.82919	CUL2 (cullin 2)	8.16
TATTTTTTTA	Hs.533122	SFRS10 (splicing factor, arginine/serine-rich 10 [transformer 2 homolog, Drosophila])	8.16
TGACTAATAA	Hs.77867	ADORA1 (adenosine A1 receptor)	8.16

To validate our SAGE results, we performed qRT-PCR of several genes listed in Table 1 and Table S1 (*DUSP6*, *PODXL*, *PTPRZ1*, *FABP7*, *FAM70A*, and *MAN1C1*) in the original line utilized for the SAGE analysis as well as in two additional GBM stem-like cell lines before and after differentiation. We confirmed the change (loss or gain) of expression of these genes upon differentiation. The only exception was *FABP7*, which exhibited no change in expression upon differentiation in HSR-GBM3; in the other lines, a loss of expression was observed (Table S2).

To confirm PODXL expression is altered with GBM stem-like cell differentiation, we performed analytical flow cytometry for PODXL on undifferentiated and differentiated cells from an additional GBM stem-like cell line, JHU-0879. The cell surface expression of PODXL was higher in undifferentiated cells compared to differentiated cells, at 34.32% and 0.42%, respectively (Figure S2A–B), confirming the SAGE and qRT-PCR results. Analysis of CD133 was included as a control and its expression was higher in undifferentiated cells compared to differentiated cells, at 87.22% and 2.63%, respectively (Figure S2C–D). Given the increased expression of PODXL in undifferentiated GBM oncospheres and its high expression in hESC, we chose to further characterize this protein in GBMs.

### PODXL expression in GBM stem-like cells

**Figure 1:**
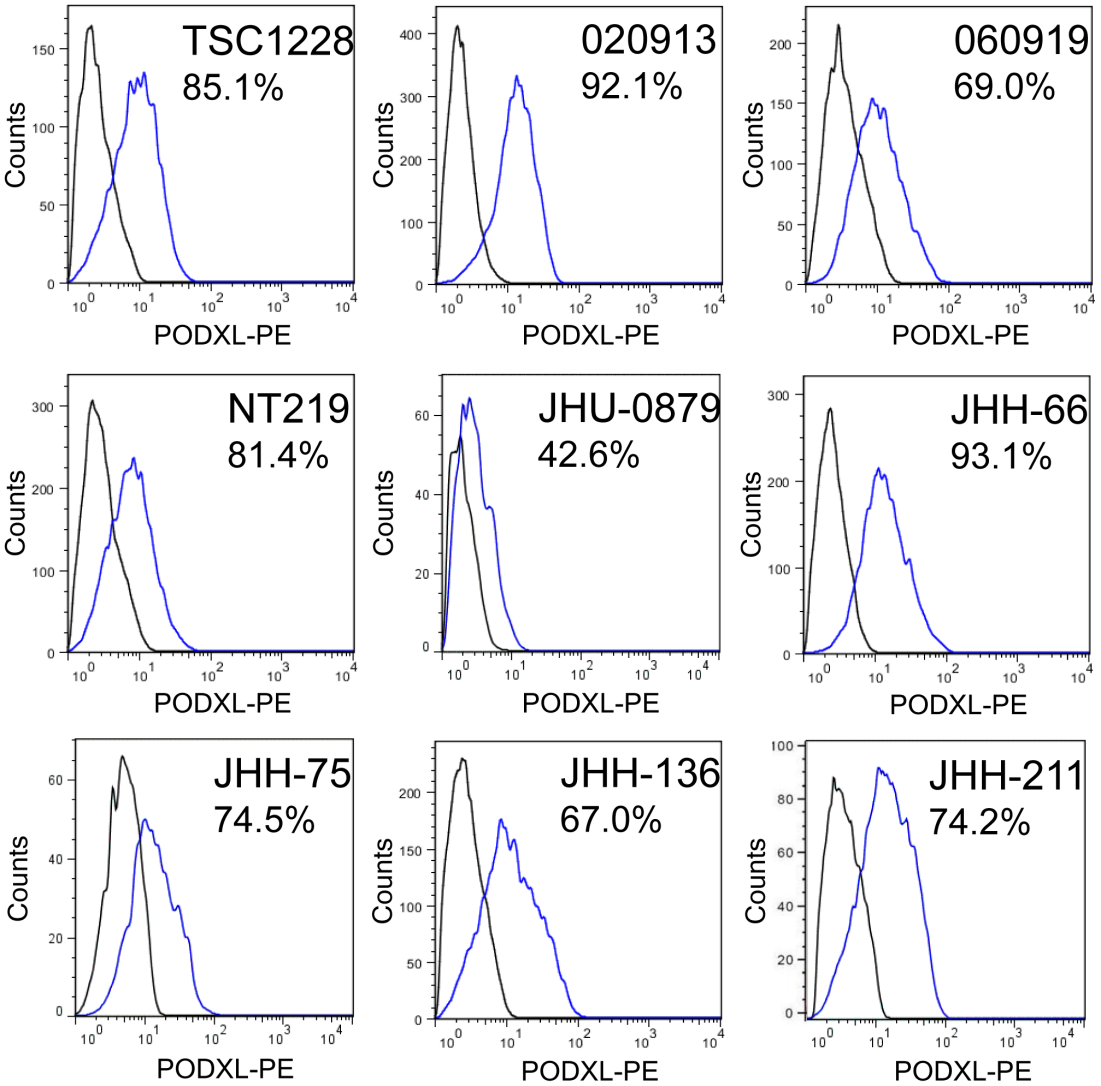
Analytical flow cytometry of GBM oncosphere lines for PODXL. All GBM oncosphere cell lines tested expressed PODXL, with the majority of the cell lines showing moderate expression of PODXL. The numbers represent the percent of cells positive for PODXL. The gate was set using 1.0% positive events from the PE-isotype population. The percent positive ranged from 42.6% to 93.1%.

We investigated the expression of PODXL in GBM oncosphere lines by analytical flow cytometry on 3 previously established (020913, 060919, and TSC1228) and numerous newly derived GBM stem-like cell lines. All of the lines examined expressed some degree of PODXL and the expression ranged from 42.6% to 93.1% of the cell population (Figure 1). For subsequent functional studies, we chose two GBM oncosphere lines, 020913 and JHU-0879.

### PODXL is a marker of GBM stem-like cell proliferation

**Figure 2:**
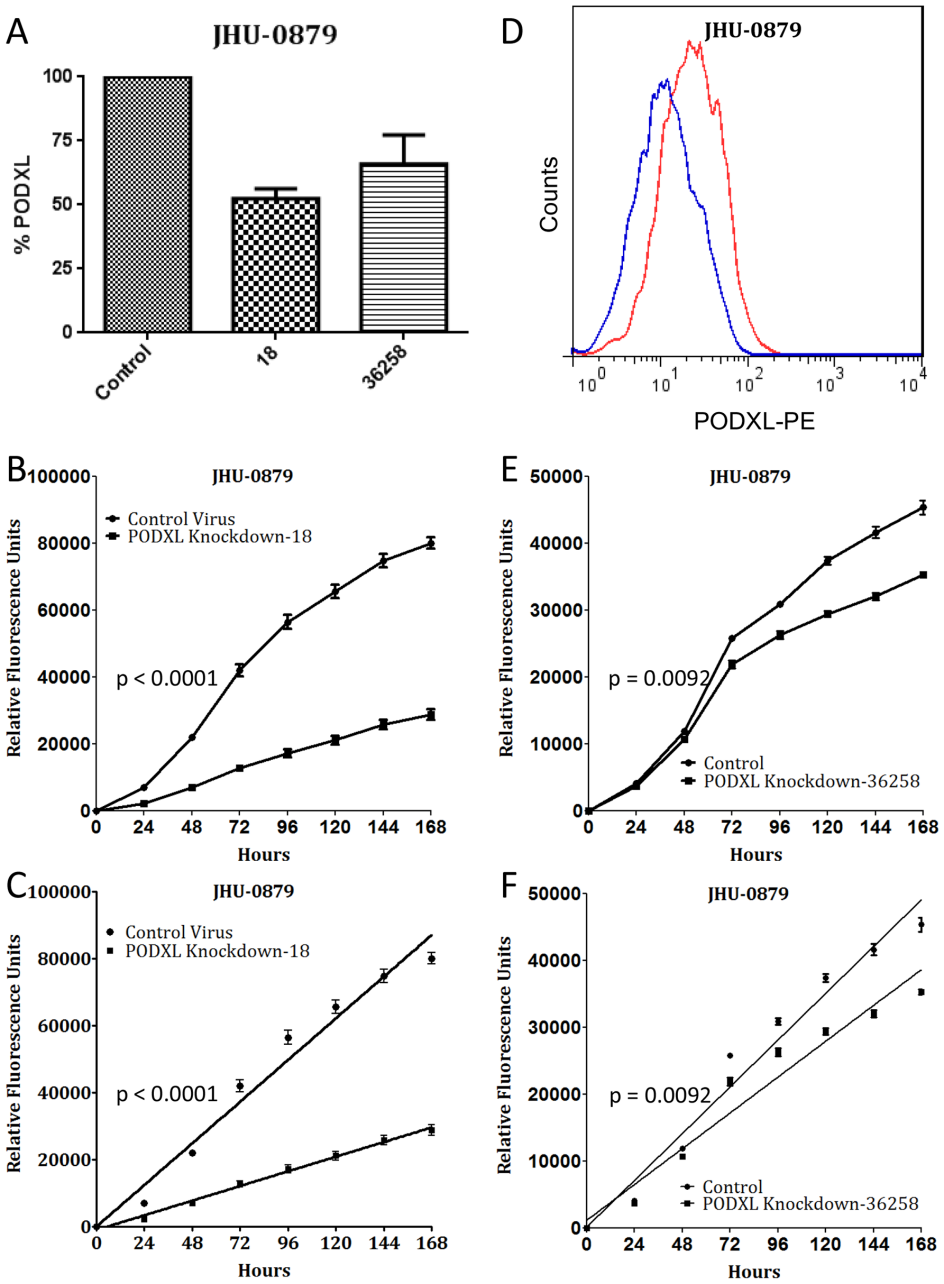
Knockdown of PODXL expression correlates with decreased cell proliferation. (A): Lentiviral knockdown of PODXL expression, as assayed by flow cytometry, using the two shRNA sequences utilized for functional studies. (B): Representative growth curve of PODXL knockdown with TRCN0000117018 and control knockdown in JHU-0879 cells over 7 days and (C) the linear regression fit of the growth curves (p<0.0001). Quantitatively similar results were obtained with each replicate. (D): Analytical flow cytometry histogram of JHU-0879 cells transduced with either control virus (red) or TRCN0000117018 virus (blue), demonstrating the knockdown of PODXL expression. (E): Representative growth curve of PODXL knockdown with V2THS_36258 and control knockdown in JHU-0879 cells over 7 days and (F) the linear regression fit of the growth curves (p = 0.0092). Quantitatively similar results were obtained with each replicate.

We next evaluated the functional role of PODXL in GBM stem-like cell proliferation, utilizing both knockdown studies and FACS. Initially, we evaluated five shRNA lentiviral vectors and chose the one with the strongest reduction of PODXL expression, TRCN0000117018 (Figure 2A and D). JHU-0879 cells were then transduced with either the PODXL knockdown lentivirus or an empty control lentivirus. The control cells grew significantly faster than the PODXL knockdown cells (p<0.0001, Figure 2B–C).

To confirm the results were due to the knockdown of PODXL and not off-target effects of the lentiviral insertion, a second shRNA screen was performed with a panel of inducible lentiviruses. Selecting the sequence with the strongest reduction of PODXL expression, V2THS_36258 (Figure 2A), proliferation assays were again performed in comparison to an empty control lentivirus. The control cells grew significantly faster than the PODXL knockdown cells (p = 0.0092, Figure 2E–F).

**Figure 3:**
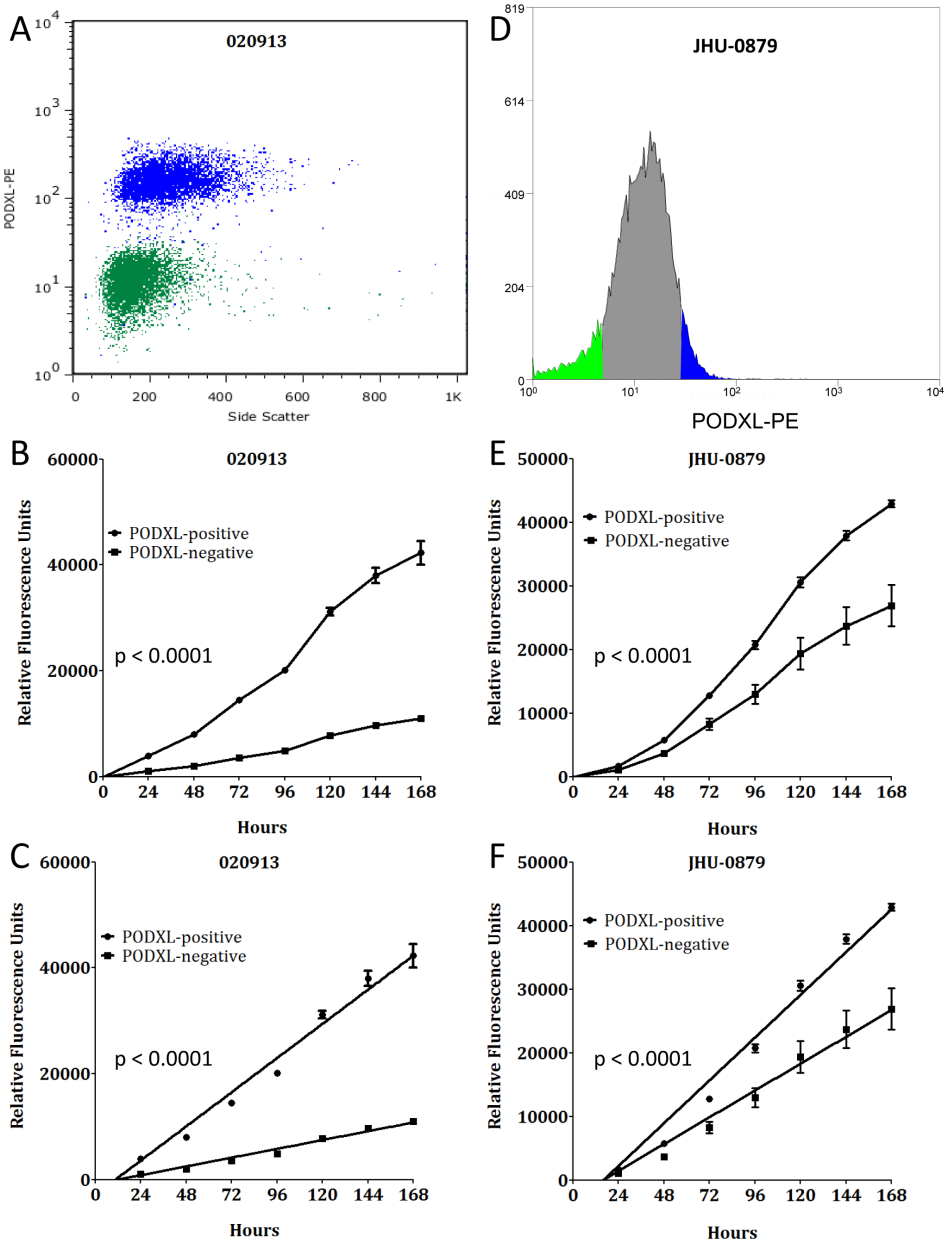
PODXL expression correlates with cell proliferation. (A): Representative post-sort analytical flow for PODXL-positive (blue) and PODXL-negative (green) populations in 020913 cells showing minimal overlap between the two populations. (B): Representative growth curves of the PODXL-positive and PODXL-negative populations from 020913 over 7 days and (C) the linear regression fit of the growth curve through day 7 (p<0.0001). Quantitatively similar results were obtained with each replicate. (D): Histogram of JHU-0879 FACS showing the gated PODXL-positive (blue) and PODXL-negative (green) populations. (E): Representative growth curves of the PODXL-positive and PODXL-negative populations from JHU-0879 over 7 days and (F) the linear regression fit of the growth curve through day 7 (p<0.0001). Quantitatively similar results were obtained with each replicate.

We then examined the role of PODXL in GBM stem-like cell proliferation by using FACS. Two independent GBM oncosphere lines, 020913 and JHU-0879, were sorted for the presence or absence of PODXL (Figure 3A, D). In each case, sort purity exceeded 95%. For each cell line, the PODXL-positive population of cells had a significantly faster growth rate compared to the PODXL-negative population (p<0.0001, Figure 3B–C, E-F). Taken together, these results demonstrate that PODXL is involved in proliferation of GBM stem-like cell lines.

### PODXL expression is associated with oncosphere formation

**Figure 4:**
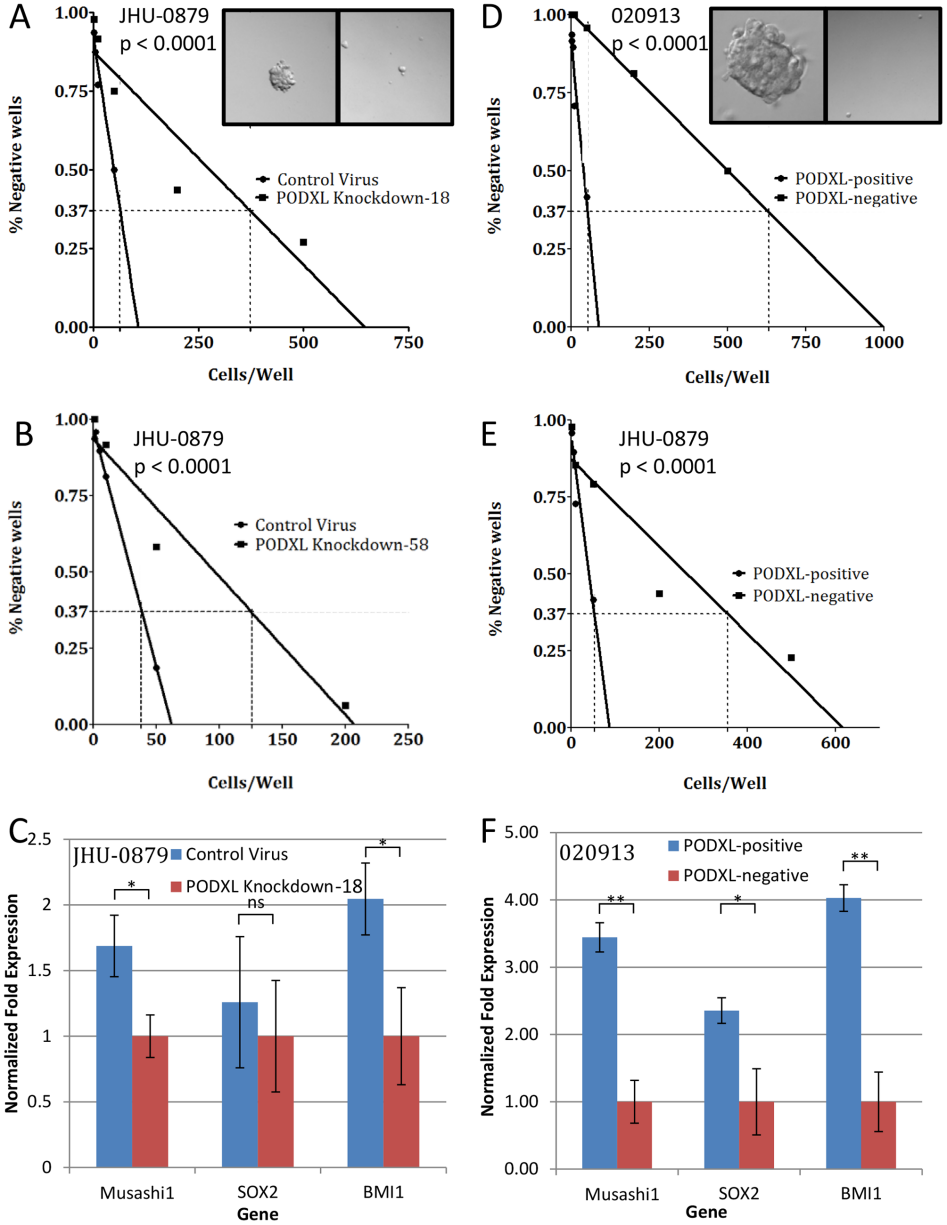
PODXL expression correlates with oncosphere formation. (A): Results of the limiting dilution assay using PODXL knockdown with TRCN0000117018 compared to control virus in JHU-0879. The 0.37 intercept was 64 cells/well for the control cells and 374 cells/well for the PODXL knockdown cells. Inset shows a control well, left, and a PODXL knockdown well, right (40×). Quantitatively similar results were obtained with two additional knockdown experiments. (B): Results of the limiting dilution assay using PODXL knockdown with V2THS_36258 compared to control virus in JHU-0879. The 0.37 intercept was 38 cells/well for the control cells and 125 cells/well for the PODXL knockdown cells. Quantitatively similar results were obtained with two additional knockdown experiments. (C): qRT-PCR results of Musashi1, SOX2, and BMI1 gene expression in JHU-0879 cells transduced with either PODXL shRNA or control virus. Expression was normalized to GAPDH expression. ns: p>0.05; *: p≤0.01. (D): Results of the limiting dilution assay using PODXL-positive cells compared to PODXL-negative cells obtained using FACS on 020913. The 0.37 intercept was 37 cells/well for the PODXL-positive cells and 632 cells/well for the PODXL-negative cells. Inset shows a PODXL-positive well, left, and a PODXL-negative well, right (40×). Quantitatively similar results were obtained with two additional FACS experiments using 020913. (E): Results of the limiting dilution assay using PODXL-positive cells compared to PODXL-negative cells obtained using FACS on JHU-0879. The 0.37 intercept was 53 cells/well for the PODXL-positive cells and 356 cells/well for the PODXL-negative cells. Quantitatively similar results were obtained with two additional FACS experiments using JHU-0879. (F): qRT-PCR results of Musashi1, SOX2, and BMI1 gene expression comparing FACS-sorted PODXL-positive and PODXL-negative populations from 020913. Expression was normalized to GAPDH expression. *: p≤0.01; **: p≤0.001.

We next investigated the role of PODXL in oncosphere formation. Our initial studies compared cells transduced with the control lentivirus to cells transduced with the PODXL-knockdown shRNA lentivirus TRCN0000117018 in JHU-0879. The percent of wells without oncospheres was graphed against the concentration of cells. In the PODXL-positive population, 1∶64 cells (1.56%) were oncosphere initiating cells whereas in the PODXL-negative population, 1∶374 cells (0.27%) were oncosphere initiating cells (p<0.0001, Figure 4A). Similar results were obtained with the inducible lentivirus, V2THS_36258, which demonstrated 1∶38 cells (2.63%) were oncosphere initiating cells in the PODXL-positive population compared to 1∶125 cells (0.80%) in the PODXL-negative population (p<0.0001, Figure 4B).

We next carried out this experiment with sorted PODXL-positive and PODXL-negative populations in both 020913 and JHU-0879 (Figure 4D and E). In 029013, this assay revealed that for the PODXL-positive population, 1∶37 cells (2.70%) were oncosphere initiating cells while in the PODXL-negative population, 1∶632 cells (0.16%) were oncosphere initiating cells (p<0.0001). This showed a greater than 10-fold enrichment in the PODXL-positive population with oncosphere-initiating cells (Figure 4D). In JHU-0879, 1∶53 cells (1.89%) in the PODXL-positive population were oncosphere initiating cells while 1∶356 cells (0.28%) in the PODXL-negative population were oncosphere initiating cells. Spheres from positive wells were replated as individual cells and were able to form new oncospheres and could be differentiated into multiple lineages (data not shown). Taken together, these studies demonstrate that PODXL expression correlates with oncosphere formation.

### PODXL expression correlates with other stem cell markers

We next chose to investigate the expression of the progenitor/stem cell markers, Musashi1, SOX2 and BMI1. JHU-0879 cells were transduced with either an empty control vector or a PODXL-knockdown vector and these genes analyzed by qRT-PCR. Expression of Musashi1, SOX2, and BMI1 was increased 1.7-, 1.3-, and 2.0-fold, respectively, compared to control cells (Figure 4C, p = 0.003,  = 0.303, and  = 0.0058, respectively). To validate these findings, similar analysis was performed with 020913 cells sorted into PODXL-positive and PODXL-negative populations. Expression of Musashi1, SOX2, and BMI-1 was increased 3.4-, 2.4-, and 4-fold, respectively, in the PODXL-position population (Figure 4F, p<0.0001,  = 0.0015, and  = 0.0002, respectively). JHU-0879 was also sorted into PODXL positive and negative populations and demonstrated similar changes in these genes (data not shown). These data demonstrate that cells expressing PODXL contain markers of early progenitor/stem cells.

### Clinical implications of PODXL expression in human gliomas

**Figure 5:**
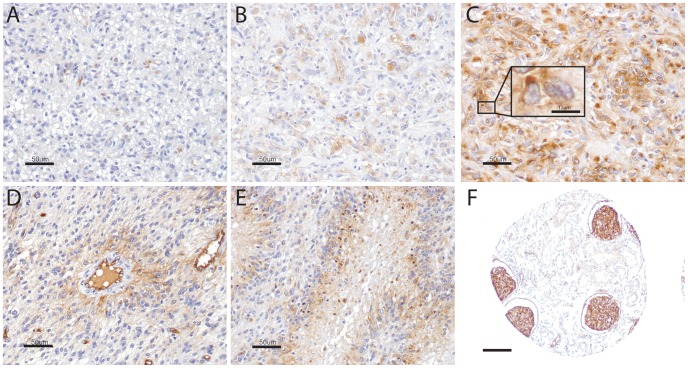
Immunohistochemical staining for PODXL expression in GBMs. Immunohistochemical staining for PODXL revealed (A) negative, (B) weak, or (C) strong levels of expression of PODXL. (C): Inset demonstrates positive staining in viable peri-necrotic cells. Tumor cells in (D) perivascular regions and (E) around necrotic foci often contained higher levels of PODXL expression. (F): Renal glomeruli served as a positive control (A–E scale bar = 50 µm, C inset scale bar = 12 µm, F scale bar = 25 µm).

We next chose to examine expression of PODXL in GBM tissue samples. Initially, eight independent GBM samples were evaluated for PODXL by immunohistochemistry. Of these eight specimens, one was negative for PODXL (Figure 5A), four showed weak PODXL staining (Figure 5B), and three showed strong PODXL staining (Figure 5C). Of the samples positive for PODXL, staining was diffuse throughout the tumor. Interestingly, in several samples, PODXL was differentially expressed in the tumor and PODXL staining was highest in the perivascular regions (Figure 5D) as well as in the pseudopalisading cells surrounding the necrotic areas (Figure 5E). PODXL immunoreactivity was present within necrotic foci, which may represent an artifact, but positively stained viable cells were also found in the pseudopalisading regions around the necrotic areas and also further away from regions of cell death. Endothelial cells within the tumor showed moderate to strong staining.

**Figure 6:**
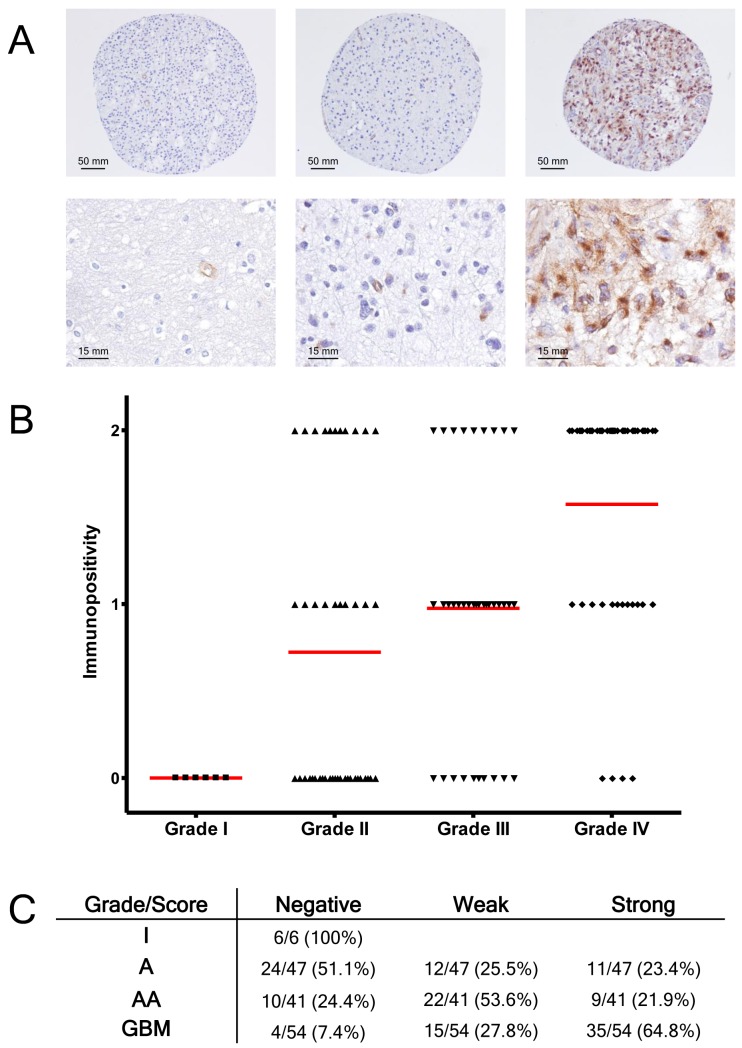
Immunohistochemical analysis of PODXL expression in gliomas. (A): Immunohistochemical staining for PODXL revealed negative (left column), weak (middle column), or strong (right column) levels of expression of PODXL (upper row 40× magnification, lower row 200× magnification). (B): Graphical representation of PODXL expression in tumor cells versus glioma grade. Red lines indicate median immunopositivity. (C): Table of PODXL immunopositivity versus grade of astrocytoma.

To further investigate the expression of PODXL in human gliomas, we examined the relationship between PODXL expression and tumor grade by performing immunohistochemistry on TMAs containing a total of 148 gliomas. We observed varying degrees of cytoplasmic and cell membrane staining (Figure 6A). Higher grade tumors had higher levels of immunoreactivity and there was a statistically significant association between PODXL expression and glioma grade (p<0.0001, Figure 6B–C).

**Figure 7:**
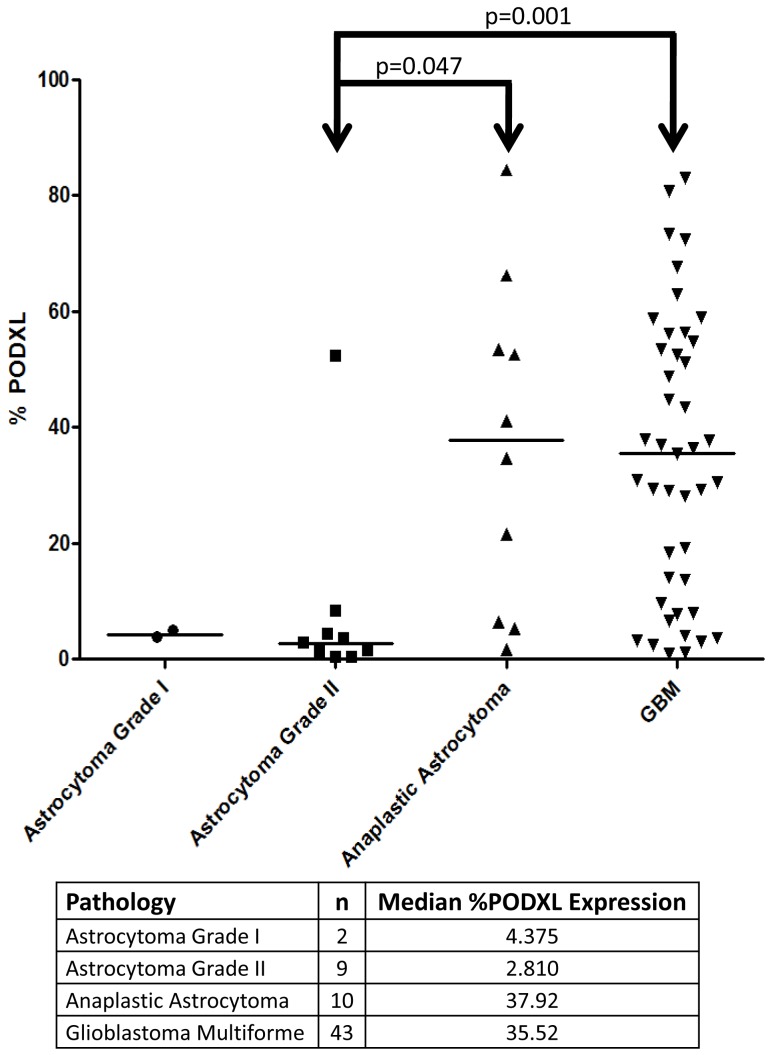
Correlation of level of PODXL expression with tumor grade. Cells were obtained from fresh tumor tissue samples and analyzed by flow cytometry for the fraction of PODXL expressing cells. There is a statistically significant difference in expression between Grade II astrocytomas and GBMs (Rank-sum test, p = 0.001), and between Grade II astrocytomas and Grade III anaplastic astrocytomas (Rank-sum test, p = 0.047).

We next examined the relationship of PODXL expression with astrocytoma grade on a total of 64 freshly resected gliomas. Tumor specimens were obtained directly from the operating room and analyzed, using flow cytometry, for the expression of PODXL. Using a Rank-sum test, we determined that there was a statistically significant difference in expression between grade II astrocytomas and GBMs (p = 0.001), and between grade II astrocytomas and grade III anaplastic astrocytomas (p = 0.047) (Figure 7). Taken together, these findings demonstrate that PODXL is expressed in primary human GBMs and that its expression level correlates positively with glioma grade.

**Figure 8:**
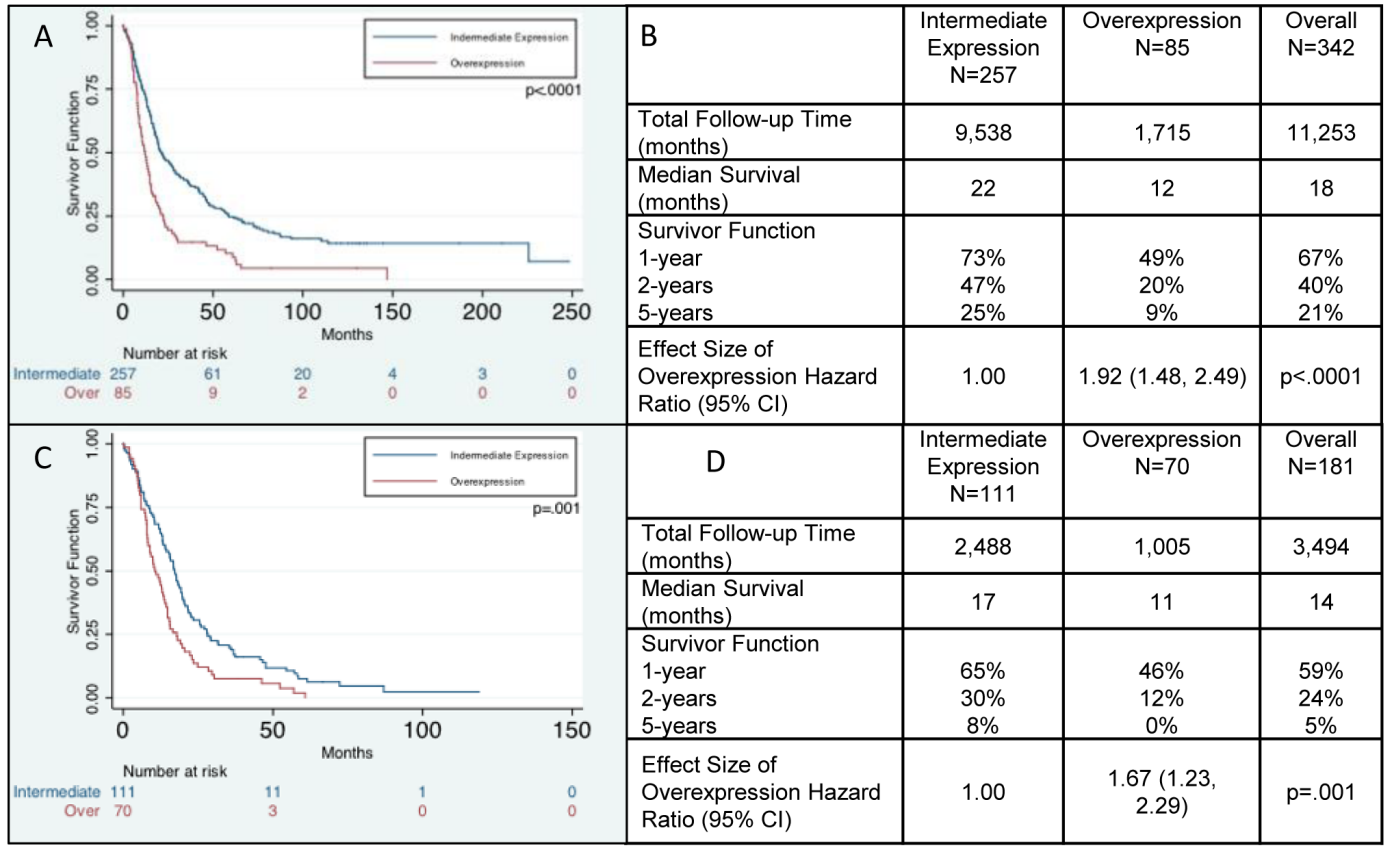
Analysis of REMBRANDT data. (A): Kaplan-Meier survival curves showing overall survival of glioma patients with high PODXL expression is shorter than glioma patients with intermediate PODXL expression (p<0.0001). (B): Median survival in months and hazard ratios of up-regulated expression of PODXL compared to intermediate expression of PODXL in all glioma patients. (C): Kaplan-Meier survival curve for GBM patients only, showing overall survival of patients with high PODXL expression is shorter than that of patients with intermediate PODXL expression (p = 0.001). (D): Median survival in months and hazard ratios of up-regulated expression of PODXL compared to intermediate expression of PODXL in GBM patients only.

We next examined the relationship between PODXL gene expression and patient outcome using the REMBRANDT data set [27]. PODXL expression was up-regulated in 85 gliomas and expressed at an intermediate level in 257 gliomas (Table S3). Patients with gliomas and up-regulated PODXL expression had a significantly shorter overall survival than those with intermediate PODXL expression (p<0.0001) (Figure 8A). The median overall survival of patients with up-regulated PODXL expression was 12 months, compared to a median overall survival of 22 months in patients with intermediate PODXL expression (Hazard ratio of 1.92, p<0.0001) (Figure 8B). This analysis contains patients with all glioma grades; we next evaluated only patients with GBM. GBM patients with up-regulated PODXL expression had a significantly shorter overall survival than patients with intermediate expression (p = 0.0003) (Figure 8C). The median overall survival of patients with up-regulated PODXL expression was 11 months, compared to a median overall survival of 17 months in patients with intermediate PODXL expression (Hazard ratio of 1.67, p = 0.001) (Figure 8D). Taken together, these studies demonstrate that PODXL expression is correlated with glioma grade and that PODXL is a marker for overall worse survival in patients with GBMs.

## Discussion

In this study, we performed transcript profiling of a GBM oncosphere line before and after differentiation. One of the notable genes up-regulated in the undifferentiated oncospheres was PODXL. As this gene is highly expressed in hESC [31]–[37] and other human malignancies [38]–[54], this was the focus of the current studies. Our findings demonstrate that PODXL is involved in GBM stem-like cell proliferation and oncosphere formation and that high PODXL expression correlates with glioma grade and decreased overall survival in patients with GBMs.

PODXL is a cell surface glycoprotein which is closely related to CD34 and endoglycan. It is physiologically expressed by kidney podocytes, hematopoietic progenitors, and vascular endothelia [47]. This protein was first identified as a 140 kDa cell surface protein on renal glomerular epithelial cells [55]; *Podxl^−/−^* mice died of anuric renal failure within 24 hours of birth due to lack of filtration slits between renal glomeruli foot processes and formation of tight junctions between adjacent podocytes [56]. Subsequent studies have demonstrated that PODXL plays a role in cell adhesion [47], [56]–[59].

Our SAGE analysis, qRT-PCR, and flow cytometric results demonstrated that PODXL is overexpressed in undifferentiated oncospheres compared to their differentiated progeny. Furthermore, bioinformatic analysis of SAGE transcript profiles [23] revealed that PODXL was highly expressed in hESCs. This is consistent with several previous studies which have demonstrated high expression of PODXL in hESCs [31]–[37] and that PODXL expression is lost as cells began to differentiate [32], [35]. The association of PODXL with hESCs is significant, as a recent paper demonstrated preferential overexpression of hESC markers in poorly differentiated cancers, including GBM [60].

PODXL has also been found in adult stem cell populations. PODXL has been shown to be a marker for early progenitor cells for multipotent mesenchymal stromal cells (MSCs) [61]. Additional studies have identified PODXL on human and murine progenitor hematopoietic stem cells, but not on mature blood cells [34], [43]. These reports further support a role for PODXL in stem cell populations.

With respect to the brain, PODXL has been found in developing and adult brain. PODXL expression was highest in the postnatal murine cerebellum, cortical plate, and hippocampus [62], the latter containing a niche for neural stem cells. Functional analysis of PODXL in normal mouse brain showed loss of PODXL resulted in increased ventricular size and intraventricular capillary formation [63] as well as decreased axonal elongation, branching, and synaptogenesis [64]. PODXL was shown to be expressed in migrating cells in the developing cerebellum and proposed to participate in neuronal migration and detachment of migrating neurons from radial glia [65].

Taken together, these previous studies support a role for PODXL as a marker for hESC, MSCs, progenitor hematopoietic stem cells, and developing brain tissue. Our data, which demonstrate that PODXL is a marker of undifferentiated GBM stem-like cells, are consistent with these findings. A recent study using lectin microarrays found six glycoproteins, including PODXL, to be differentially expressed between the stem-like oncosphere line 020913 and the traditional adherent line U373 [15].

We examined the function of PODXL in GBM stem-like cells and demonstrated that PODXL is involved in proliferation. Both viral knockdown studies and cell sorting experiments showed that PODXL positive cells grow significantly faster than PODXL negative cells. We did notice a smaller difference in growth rates between V2THS_36258 PODXL knockdown and its control virus than between TRCN0000117018 and its control virus. We feel this is most likely due the decreased level of PODXL knockdown achieved with V2THS_36258, as shown in Figure 2A. To the best of our knowledge, this is the first demonstration of a direct functional role of PODXL in cell proliferation.

In addition to establishing a role in GBM stem-like cell proliferation, our results demonstrated that PODXL is involved in oncosphere formation. Both viral knockdown assays and cell sorting experiments demonstrated that PODXL positive cells are able to form oncospheres in a limiting dilution assay. The determined concentration of PODXL-positive cells required to form an oncosphere was significantly lower than that of the PODXL-negative cells, demonstrating that the PODXL-positive population contains more cells capable of clonal replication. Although the mechanism(s) remains to be established, this does not appear to involve apoptosis as PODXL shRNA knockdown studies and FACS analysis show no statistical difference in caspase activity between these two populations (Figure S3).

The role of PODXL as a marker for undifferentiated GBM stem-like cells is further supported by the increased expression of the early progenitor/stem cell markers Musashi1, SOX2, and BMI1 in PODXL positive cells [66]. We evaluated the expression levels of these markers and found them to be increased in the PODXL-positive cells compared to the PODXL-negative cells. Taken together, these results demonstrate that PODXL is involved in GBM stem-like cell proliferation, oncosphere formation, and marks cells with increased early progenitor/stem cell marker expression.

We additionally examined the expression of PODXL in gliomas and demonstrated that PODXL is overexpressed in the majority of GBMs and that PODXL expression is correlated with tumor grade, demonstrated in both fixed specimens and fresh tissue. This is consistent with previous findings of PODXL over-expression in a variety of other cancers, including leukemias [42], [43], pancreatic ductal adenocarcinoma [46], invasive breast carcinomas [50], prostate cancer [67], Wilms tumor [51], hepatocellular carcinoma [40], embryonal carcinoma (EC) [48], [49], small cell lung carcinoma (SCLC) [44], undifferentiated thyroid carcinomas [52], colorectal cancers [45], renal cell carcinoma [41], endometrial adenocarcinoma [53], ovarian carcinomas [54], and GBM [39]. Mutations affecting the extracellular domain of PODXL are associated with an increased risk of prostate cancer and development of a more aggressive prostate cancer [38]. Overexpression of PODXL has been reported to correlate with a more aggressive phenotype of breast cancer [67], prostate cancer [67], and renal cell carcinoma [41]. A number of other studies have demonstrated an inverse correlation between PODXL expression and degree of tumor differentiation. This has been observed in breast cancers [50], GBMs [39], and thyroid cancers [52]. PODXL has also been shown to be expressed in several cancers with stem-like cell populations [44], [48]. In EC, PODXL was shown to be a marker of undifferentiated EC cells when compared with EC cells forced to differentiate by retinoic acid [48]. Additionally, in SCLC, PODXL was found to be co-expressed with BMI1 in multipotent precursor cells [44].

Our analysis, utilizing the REMBRANDT data set, demonstrated that up-regulation of PODXL predicts worse survival for patients with glioma. Of even greater significance is that, within the GBM cohort alone, tumors with up-regulated PODXL expression were associated with a significantly shorter overall survival. One important consideration in this analysis is the lack of specificity of cell type in the REMBRANDT data set. Our data and the work of others [68] demonstrate that PODXL is expressed in endothelium. As such, the REMBRANDT data reflects PODXL staining in both tumor cells and also the associated vasculature in the sample. Although this is a potential confounding variable, the amount of PODXL expressed in vessels in our immunohistochemical studies (Figures 5 and 6) was a minor component and thus we feel the survival curves are primarily reflective of expression in the tumors cells.

In addition to PODXL's involvement in cell adhesion, it has been demonstrated to play a role in invasion. PODXL has been shown to increase the invasive and migratory potential of MCF7 breast cancer and PC3 prostate cancer cells *in vitro* and leads to an increase in mitogen-activated protein kinase and phosphatidylinositol 3-kinase activity [67]. During review of this manuscript, an additional study investigating the role of PODXL in glioma cell invasion was published, demonstrating that expression of PODXL increases *in vitro* invasion and protects against chemotherapeutic activity [69]. Additional studies will be necessary to further explore the role of PODXL in glioma invasion.

## Conclusion

In this study, we demonstrate PODXL is expressed in undifferentiated GBM stem-like cells. PODXL expression correlates with cellular proliferation and oncosphere formation and PODXL-positive cells express higher levels of stem cell markers compared to their PODXL-negative counterparts. Finally, high PODXL expression correlates with glioma grade and poorer outcome in patients with GBM.

## Supporting Information

Figure S1 **SAGE Genie bioinformatic analysis of PODXL expression.** Bioinformatic analyses of transcript expression data revealed that PODXL is highly expressed in human embryonic stem cell lines as well as human embryonal carcinoma cell lines (http://cgap.nci.nih.gov/SAGE).
(TIF)

Figure S2 **Analytical flow cytometry of PODXL and CD133 expression in undifferentiated and differentiated JHU-0879 cells.** Expression of both PODXL and CD133 decrease upon differentiation. (A): JHU-0879 cells stained with PE-PODXL antibody and PE-isotype antibody, demonstrating 22.51% positivity. (B): Differentiated JHU-0879 cells stained with PE-PODXL antibody and PE-isotype antibody, demonstrating 0.42% positivity. (C): JHU-0879 cells stained with PE-CD133 antibody and PE-isotype antibody, demonstrating 87.22% positivity. (D): Differentiated JHU-0879 cells stained with PE-CD133 antibody and PE-isotype antibody, demonstrating 2.63% positivity.
(TIF)

Figure S3 **PODXL knockdown does not induce apoptosis.** GBM stem-like cell line JHU-0879 was transduced with either shControl or shPODXL and assayed for Caspase 3/7 activity. The positive control was sunitinib at 20 µM and the negative control was wells without cells. There was no apoptosis seen with PODXL knockdown.
(TIF)

Table S1 **Genes overexpressed in differentiated GBM oncospheres.**
(DOCX)

Table S2 **Quantitative real-time PCR expression analysis in three matched sets of undifferentiated and differentiated GBM oncosphere lines.**
(DOCX)

Table S3 **Pathological diagnoses of the REMBRANDT dataset.**
(DOCX)

## References

[1] Stupp R, Mason WP, van den Bent MJ, Weller M, Fisher B, et al (2005) Radiotherapy plus concomitant and adjuvant temozolomide for glioblastoma. N Engl J Med 352: 987–996. 15758009 10.1056/NEJMoa043330

[2] Galli R, Binda E, Orfanelli U, Cipelletti B, Gritti A, et al (2004) Isolation and characterization of tumorigenic, stem-like neural precursors from human glioblastoma. Cancer Res 64: 7011–7021. 15466194 10.1158/0008-5472.CAN-04-1364

[3] Singh SK, Hawkins C, Clarke ID, Squire JA, Bayani J, et al (2004) Identification of human brain tumour initiating cells. Nature 432: 396–401. 15549107 10.1038/nature03128

[4] Bao S, Wu Q, McLendon RE, Hao Y, Shi Q, et al (2006) Glioma stem cells promote radioresistance by preferential activation of the DNA damage response. Nature 444: 756–760. 17051156 10.1038/nature05236

[5] Eramo A, Ricci-Vitiani L, Zeuner A, Pallini R, Lotti F, et al (2006) Chemotherapy resistance of glioblastoma stem cells. Cell Death Differ 13: 1238–1241. 16456578 10.1038/sj.cdd.4401872

[6] Mao XG, Zhang X, Xue XY, Guo G, Wang P, et al (2009) Brain Tumor Stem-Like Cells Identified by Neural Stem Cell Marker CD15. Transl Oncol 2: 247–257. 19956386 10.1593/tlo.09136 PMC2781066

[7] Jijiwa M, Demir H, Gupta S, Leung C, Joshi K, et al (2011) CD44v6 regulates growth of brain tumor stem cells partially through the AKT-mediated pathway. PLoS One 6: e24217. 21915300 10.1371/journal.pone.0024217 PMC3167830

[8] Bao S, Wu Q, Li Z, Sathornsumetee S, Wang H, et al (2008) Targeting cancer stem cells through L1CAM suppresses glioma growth. Cancer Res 68: 6043–6048. 18676824 10.1158/0008-5472.CAN-08-1079 PMC2739001

[9] Lathia JD, Gallagher J, Heddleston JM, Wang J, Eyler CE, et al (2010) Integrin alpha 6 regulates glioblastoma stem cells. Cell Stem Cell 6: 421–432. 20452317 10.1016/j.stem.2010.02.018 PMC2884275

[10] Beier D, Hau P, Proescholdt M, Lohmeier A, Wischhusen J, et al (2007) CD133(+) and CD133(−) glioblastoma-derived cancer stem cells show differential growth characteristics and molecular profiles. Cancer Res 67: 4010–4015. 17483311 10.1158/0008-5472.CAN-06-4180

[11] Joo KM, Kim SY, Jin X, Song SY, Kong DS, et al (2008) Clinical and biological implications of CD133-positive and CD133-negative cells in glioblastomas. Lab Invest 88: 808–815. 18560366 10.1038/labinvest.2008.57

[12] Shen G, Shen F, Shi Z, Liu W, Hu W, et al (2008) Identification of cancer stem-like cells in the C6 glioma cell line and the limitation of current identification methods. In Vitro Cell Dev Biol Anim 44: 280–289. 18594936 10.1007/s11626-008-9115-z

[13] Patru C, Romao L, Varlet P, Coulombel L, Raponi E, et al (2010) CD133, CD15/SSEA-1, CD34 or side populations do not resume tumor-initiating properties of long-term cultured cancer stem cells from human malignant glio-neuronal tumors. BMC Cancer 10: 66. 20181261 10.1186/1471-2407-10-66 PMC2841664

[14] Al-Hajj M, Wicha MS, Benito-Hernandez A, Morrison SJ, Clarke MF (2003) Prospective identification of tumorigenic breast cancer cells. Proc Natl Acad Sci U S A 100: 3983–3988. 12629218 10.1073/pnas.0530291100 PMC153034

[15] He J, Liu Y, Xie X, Zhu T, Soules M, et al (2010) Identification of cell surface glycoprotein markers for glioblastoma-derived stem-like cells using a lectin microarray and LC-MS/MS approach. J Proteome Res 9: 2565–2572. 20235609 10.1021/pr100012p PMC2866009

[16] Hirschmann-Jax C, Foster AE, Wulf GG, Nuchtern JG, Jax TW, et al (2004) A distinct “side population” of cells with high drug efflux capacity in human tumor cells. Proc Natl Acad Sci U S A 101: 14228–14233. 15381773 10.1073/pnas.0400067101 PMC521140

[17] Platet N, Mayol JF, Berger F, Herodin F, Wion D (2007) Fluctuation of the SP/non-SP phenotype in the C6 glioma cell line. FEBS Lett 581: 1435–1440. 17362939 10.1016/j.febslet.2007.02.071

[18] Lee J, Kotliarova S, Kotliarov Y, Li A, Su Q, et al (2006) Tumor stem cells derived from glioblastomas cultured in bFGF and EGF more closely mirror the phenotype and genotype of primary tumors than do serum-cultured cell lines. Cancer Cell 9: 391–403. 16697959 10.1016/j.ccr.2006.03.030

[19] Siu IM, Tyler BM, Chen JX, Eberhart CG, Thomale UW, et al (2010) Establishment of a human glioblastoma stemlike brainstem rodent tumor model. J Neurosurg Pediatr 6: 92–97. 20593994 10.3171/2010.3.PEDS09366

[20] Zhu TS, Costello MA, Talsma CE, Flack CG, Crowley JG, et al (2011) Endothelial cells create a stem cell niche in glioblastoma by providing NOTCH ligands that nurture self-renewal of cancer stem-like cells. Cancer Res 71: 6061–6072. 21788346 10.1158/0008-5472.CAN-10-4269 PMC3355476

[21] Porter DA, Krop IE, Nasser S, Sgroi D, Kaelin CM, et al (2001) A SAGE (serial analysis of gene expression) view of breast tumor progression. Cancer Res 61: 5697–5702. 11479200

[22] Boon K, Osorio EC, Greenhut SF, Schaefer CF, Shoemaker J, et al (2002) An anatomy of normal and malignant gene expression. Proc Natl Acad Sci U S A 99: 11287–11292. 12119410 10.1073/pnas.152324199 PMC123249

[23] Cancer Genome Anatomy Project (2002) SAGE Genie. Available: http://cgap.nci.nih.gov/SAGE Accessed on 2 June 2010.

[24] Cerutti JM, Delcelo R, Amadei MJ, Nakabashi C, Maciel RM, et al (2004) A preoperative diagnostic test that distinguishes benign from malignant thyroid carcinoma based on gene expression. J Clin Invest 113: 1234–1242. 15085203 10.1172/JCI19617 PMC385398

[25] Loilome W, Joshi AD, ap Rhys CM, Piccirillo S, Vescovi AL, et al (2009) Glioblastoma cell growth is suppressed by disruption of Fibroblast Growth Factor pathway signaling. J Neurooncol 94: 359–366. 19340397 10.1007/s11060-009-9885-5

[26] Siu IM, Pretlow TG, Amini SB, Pretlow TP (1997) Identification of dysplasia in human colonic aberrant crypt foci. Am J Pathol 150: 1805–1813. 9137103 PMC1858202

[27] NationalCancerInstitute (2005) REMBRANDT home page. Available: http://rembrandt.nci.nih.gov Accessed on 6 Novemeber 2010.

[28] Schmittgen TD, Livak KJ (2008) Analyzing real-time PCR data by the comparative C(T) method. Nat Protoc 3: 1101–1108. 18546601 10.1038/nprot.2008.73

[29] Tropepe V, Sibilia M, Ciruna BG, Rossant J, Wagner EF, et al (1999) Distinct neural stem cells proliferate in response to EGF and FGF in the developing mouse telencephalon. Dev Biol 208: 166–188. 10075850 10.1006/dbio.1998.9192

[30] Hu Y, Smyth GK (2009) ELDA: extreme limiting dilution analysis for comparing depleted and enriched populations in stem cell and other assays. J Immunol Methods 347: 70–78. 19567251 10.1016/j.jim.2009.06.008

[31] Bhattacharya B, Miura T, Brandenberger R, Mejido J, Luo Y, et al (2004) Gene expression in human embryonic stem cell lines: unique molecular signature. Blood 103: 2956–2964. 15070671 10.1182/blood-2003-09-3314

[32] Cai J, Chen J, Liu Y, Miura T, Luo Y, et al (2006) Assessing self-renewal and differentiation in human embryonic stem cell lines. Stem Cells 24: 516–530. 16293578 10.1634/stemcells.2005-0143 PMC1855239

[33] Cai J, Olson JM, Rao MS, Stanley M, Taylor E, et al (2005) Development of antibodies to human embryonic stem cell antigens. BMC Dev Biol 5: 26. 16316465 10.1186/1471-213X-5-26 PMC1315352

[34] Doyonnas R, Nielsen JS, Chelliah S, Drew E, Hara T, et al (2005) Podocalyxin is a CD34-related marker of murine hematopoietic stem cells and embryonic erythroid cells. Blood 105: 4170–4178. 15701716 10.1182/blood-2004-10-4077

[35] Laslett AL, Grimmond S, Gardiner B, Stamp L, Lin A, et al (2007) Transcriptional analysis of early lineage commitment in human embryonic stem cells. BMC Dev Biol 7: 12. 17335568 10.1186/1471-213X-7-12 PMC1829156

[36] Richards M, Tan SP, Tan JH, Chan WK, Bongso A (2004) The transcriptome profile of human embryonic stem cells as defined by SAGE. Stem Cells 22: 51–64. 14688391 10.1634/stemcells.22-1-51

[37] Zeng X, Miura T, Luo Y, Bhattacharya B, Condie B, et al (2004) Properties of pluripotent human embryonic stem cells BG01 and BG02. Stem Cells 22: 292–312. 15153607 10.1634/stemcells.22-3-292

[38] Casey G, Neville PJ, Liu X, Plummer SJ, Cicek MS, et al (2006) Podocalyxin variants and risk of prostate cancer and tumor aggressiveness. Hum Mol Genet 15: 735–741. 16434482 10.1093/hmg/ddi487

[39] Hayatsu N, Kaneko MK, Mishima K, Nishikawa R, Matsutani M, et al (2008) Podocalyxin expression in malignant astrocytic tumors. Biochem Biophys Res Commun 374: 394–398. 18639524 10.1016/j.bbrc.2008.07.049

[40] Heukamp LC, Fischer HP, Schirmacher P, Chen X, Breuhahn K, et al (2006) Podocalyxin-like protein 1 expression in primary hepatic tumours and tumour-like lesions. Histopathology 49: 242–247. 16918970 10.1111/j.1365-2559.2006.02489.x

[41] Hsu YH, Lin WL, Hou YT, Pu YS, Shun CT, et al (2010) Podocalyxin EBP50 ezrin molecular complex enhances the metastatic potential of renal cell carcinoma through recruiting Rac1 guanine nucleotide exchange factor ARHGEF7. Am J Pathol 176: 3050–3061. 20395446 10.2353/ajpath.2010.090539 PMC2877864

[42] Kelley TW, Huntsman D, McNagny KM, Roskelley CD, Hsi ED (2005) Podocalyxin: a marker of blasts in acute leukemia. Am J Clin Pathol 124: 134–142. 15923169 10.1309/7BHLAHHU0N4MHT7Q

[43] Kerosuo L, Juvonen E, Alitalo R, Gylling M, Kerjaschki D, et al (2004) Podocalyxin in human haematopoietic cells. Br J Haematol 124: 809–818. 15009070 10.1111/j.1365-2141.2004.04840.x

[44] Koch LK, Zhou H, Ellinger J, Biermann K, Holler T, et al (2008) Stem cell marker expression in small cell lung carcinoma and developing lung tissue. Hum Pathol 39: 1597–1605. 18656241 10.1016/j.humpath.2008.03.008

[45] Larsson A, Johansson ME, Wangefjord S, Gaber A, Nodin B, et al (2011) Overexpression of podocalyxin-like protein is an independent factor of poor prognosis in colorectal cancer. Br J Cancer 105: 666–672. 21829192 10.1038/bjc.2011.295 PMC3188928

[46] Ney JT, Zhou H, Sipos B, Buttner R, Chen X, et al (2007) Podocalyxin-like protein 1 expression is useful to differentiate pancreatic ductal adenocarcinomas from adenocarcinomas of the biliary and gastrointestinal tracts. Hum Pathol 38: 359–364. 17137615 10.1016/j.humpath.2006.08.025

[47] Nielsen JS, McNagny KM (2009) The role of podocalyxin in health and disease. J Am Soc Nephrol 20: 1669–1676. 19578008 10.1681/ASN.2008070782

[48] Schopperle WM, DeWolf WC (2007) The TRA-1-60 and TRA-1-81 human pluripotent stem cell markers are expressed on podocalyxin in embryonal carcinoma. Stem Cells 25: 723–730. 17124010 10.1634/stemcells.2005-0597

[49] Schopperle WM, Kershaw DB, DeWolf WC (2003) Human embryonal carcinoma tumor antigen, Gp200/GCTM-2, is podocalyxin. Biochem Biophys Res Commun 300: 285–290. 12504081 10.1016/s0006-291x(02)02844-9

[50] Somasiri A, Nielsen JS, Makretsov N, McCoy ML, Prentice L, et al (2004) Overexpression of the anti-adhesin podocalyxin is an independent predictor of breast cancer progression. Cancer Res 64: 5068–5073. 15289306 10.1158/0008-5472.CAN-04-0240

[51] Stanhope-Baker P, Kessler PM, Li W, Agarwal ML, Williams BR (2004) The Wilms tumor suppressor-1 target gene podocalyxin is transcriptionally repressed by p53. J Biol Chem 279: 33575–33585. 15155752 10.1074/jbc.M404787200

[52] Yasuoka H, Tsujimoto M, Hirokawa M, Tori M, Nakahara M, et al (2008) Podocalyxin expression in undifferentiated thyroid carcinomas. J Clin Pathol 61: 1228–1229. 18955579 10.1136/jcp.2008.059956

[53] Yasuoka H, Tsujimoto M, Inagaki M, Kodama R, Tsuji H, et al (2012) Clinicopathological significance of podocalyxin and phosphorylated ezrin in uterine endometrioid adenocarcinoma. J Clin Pathol 65: 399–402. 22412054 10.1136/jclinpath-2011-200359

[54] Cipollone JA, Graves ML, Kobel M, Kalloger SE, Poon T, et al (2012) The anti-adhesive mucin podocalyxin may help initiate the transperitoneal metastasis of high grade serous ovarian carcinoma. Clin Exp Metastasis 29: 239–252. 22262060 10.1007/s10585-011-9446-0

[55] Kerjaschki D, Sharkey DJ, Farquhar MG (1984) Identification and characterization of podocalyxin–the major sialoprotein of the renal glomerular epithelial cell. J Cell Biol 98: 1591–1596. 6371025 10.1083/jcb.98.4.1591 PMC2113206

[56] Doyonnas R, Kershaw DB, Duhme C, Merkens H, Chelliah S, et al (2001) Anuria, omphalocele, and perinatal lethality in mice lacking the CD34-related protein podocalyxin. J Exp Med 194: 13–27. 11435469 10.1084/jem.194.1.13 PMC2193439

[57] Larrucea S, Butta N, Arias-Salgado EG, Alonso-Martin S, Ayuso MS, et al (2008) Expression of podocalyxin enhances the adherence, migration, and intercellular communication of cells. Exp Cell Res 314: 2004–2015. 18456258 10.1016/j.yexcr.2008.03.009

[58] Larrucea S, Butta N, Rodriguez RB, Alonso-Martin S, Arias-Salgado EG, et al (2007) Podocalyxin enhances the adherence of cells to platelets. Cell Mol Life Sci 64: 2965–2974. 17922228 10.1007/s00018-007-7374-6 PMC11136142

[59] Takeda T, Go WY, Orlando RA, Farquhar MG (2000) Expression of podocalyxin inhibits cell-cell adhesion and modifies junctional properties in Madin-Darby canine kidney cells. Mol Biol Cell 11: 3219–3232. 10982412 10.1091/mbc.11.9.3219 PMC14987

[60] Ben-Porath I, Thomson MW, Carey VJ, Ge R, Bell GW, et al (2008) An embryonic stem cell-like gene expression signature in poorly differentiated aggressive human tumors. Nat Genet 40: 499–507. 18443585 10.1038/ng.127 PMC2912221

[61] Lee RH, Seo MJ, Pulin AA, Gregory CA, Ylostalo J, et al (2009) The CD34-like protein PODXL and alpha6-integrin (CD49f) identify early progenitor MSCs with increased clonogenicity and migration to infarcted heart in mice. Blood 113: 816–826. 18818395 10.1182/blood-2007-12-128702 PMC2630267

[62] Vitureira N, McNagny K, Soriano E, Burgaya F (2005) Pattern of expression of the podocalyxin gene in the mouse brain during development. Gene Expr Patterns 5: 349–354. 15661640 10.1016/j.modgep.2004.10.002

[63] Nowakowski A, Alonso-Martin S, Gonzalez-Manchon C, Larrucea S, Fernandez D, et al (2010) Ventricular enlargement associated with the panneural ablation of the podocalyxin gene. Mol Cell Neurosci 43: 90–97. 19837166 10.1016/j.mcn.2009.09.011

[64] Vitureira N, Andres R, Perez-Martinez E, Martinez A, Bribian A, et al (2010) Podocalyxin is a novel polysialylated neural adhesion protein with multiple roles in neural development and synapse formation. PLoS One 5: e12003. 20706633 10.1371/journal.pone.0012003 PMC2919383

[65] Garcia-Frigola C, Burgaya F, Calbet M, Lopez-Domenech G, de Lecea L, et al (2004) A collection of cDNAs enriched in upper cortical layers of the embryonic mouse brain. Brain Res Mol Brain Res 122: 133–150. 15010206 10.1016/j.molbrainres.2003.12.014

[66] Nicolis SK (2007) Cancer stem cells and “stemness” genes in neuro-oncology. Neurobiol Dis 25: 217–229. 17141509 10.1016/j.nbd.2006.08.022

[67] Sizemore S, Cicek M, Sizemore N, Ng KP, Casey G (2007) Podocalyxin increases the aggressive phenotype of breast and prostate cancer cells in vitro through its interaction with ezrin. Cancer Res 67: 6183–6191. 17616675 10.1158/0008-5472.CAN-06-3575

[68] Horvat R, Hovorka A, Dekan G, Poczewski H, Kerjaschki D (1986) Endothelial cell membranes contain podocalyxin–the major sialoprotein of visceral glomerular epithelial cells. J Cell Biol 102: 484–491. 3511072 10.1083/jcb.102.2.484 PMC2114082

[69] Wu H, Yang L, Liao D, Chen Y, Wang W, et al (2013) Podocalyxin regulates astrocytoma cell invasion and survival against temozolomide. Exp Ther Med 5: 1025–1029. 23596468 10.3892/etm.2013.957 PMC3627468

