# Supplementary figures and images for: Podocalyxin-Like Protein Is Expressed in Glioblastoma Multiforme Stem-Like Cells and Is Associated with Poor Outcome
Source: PLoS One. 2013 Oct 16;8(10):e75945. doi: 10.1371/journal.pone.0075945 (PMC3797817; doi:10.1371/journal.pone.0075945)

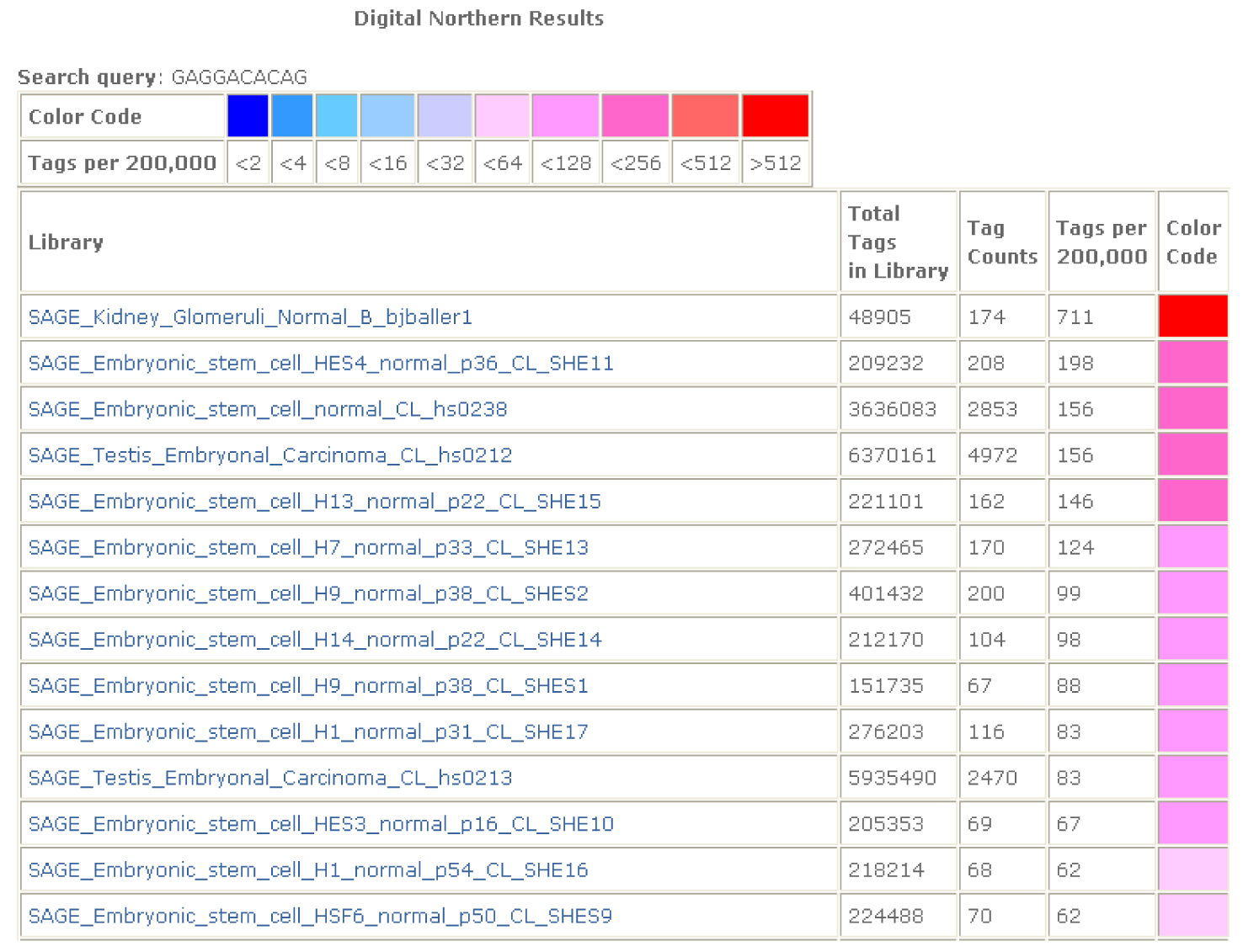

Supplement: Figure S1 — SAGE Genie bioinformatic analysis of PODXL expression. Bioinformatic analyses of transcript expression data revealed that PODXL is highly expressed in human embryonic stem cell lines as well as human embryonal carcinoma cell lines (http://cgap.nci.nih.gov/SAGE). (TIF) [file pone.0075945.s001.tif]

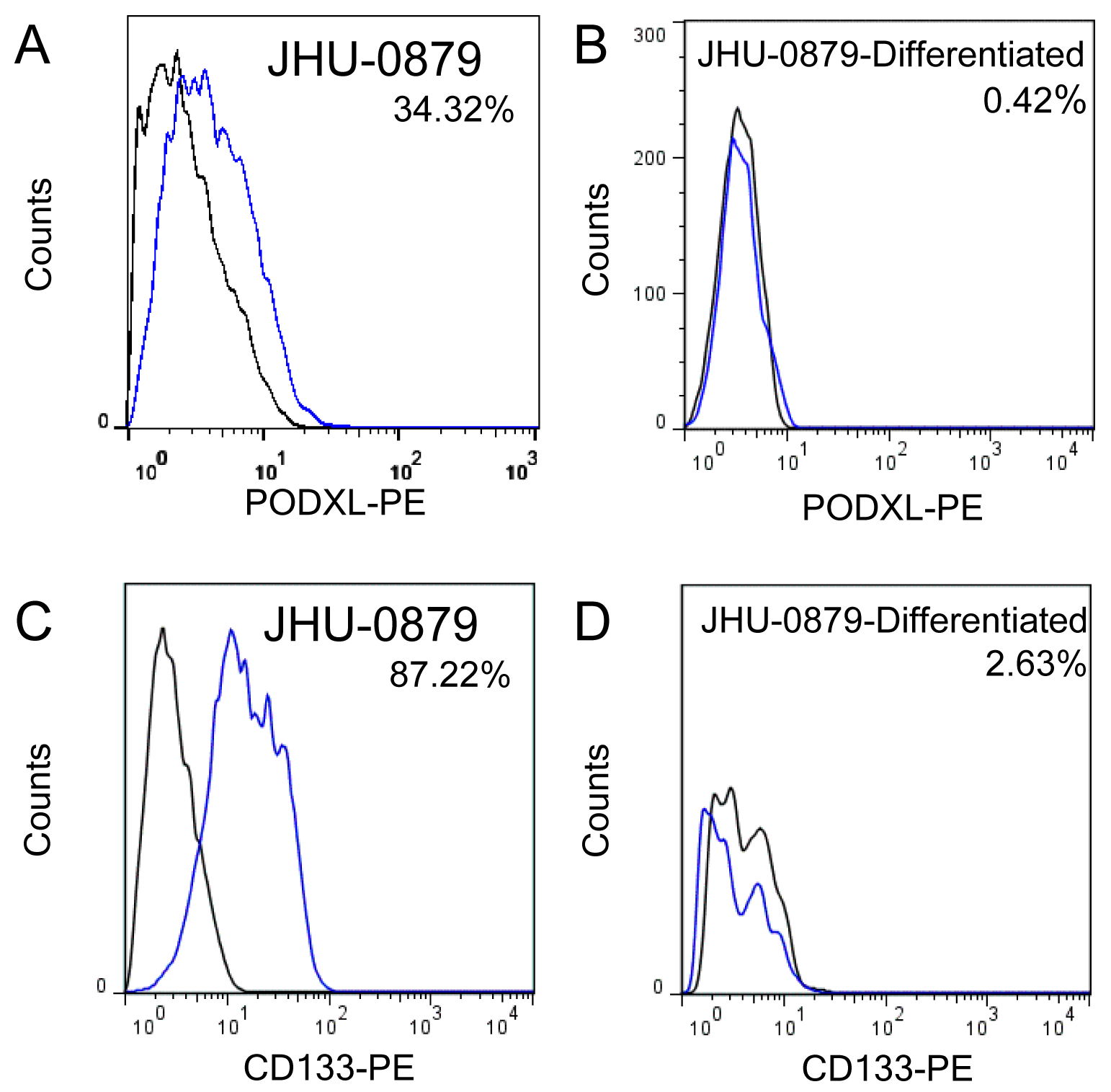

Supplement: Figure S2 — Analytical flow cytometry of PODXL and CD133 expression in undifferentiated and differentiated JHU-0879 cells. Expression of both PODXL and CD133 decrease upon differentiation. (A): JHU-0879 cells stained with PE-PODXL antibody and PE-isotype antibody, demonstrating 22.51% positivity. (B): Differentiated JHU-0879 cells stained with PE-PODXL antibody and PE-isotype antibody, demonstrating 0.42% positivity. (C): JHU-0879 cells stained with PE-CD133 antibody and PE-isotype antibody, demonstrating 87.22% positivity. (D): Differentiated JHU-0879 cells stained with PE-CD133 antibody and PE-isotype antibody, demonstrating 2.63% positivity. (TIF) [file pone.0075945.s002.tif]

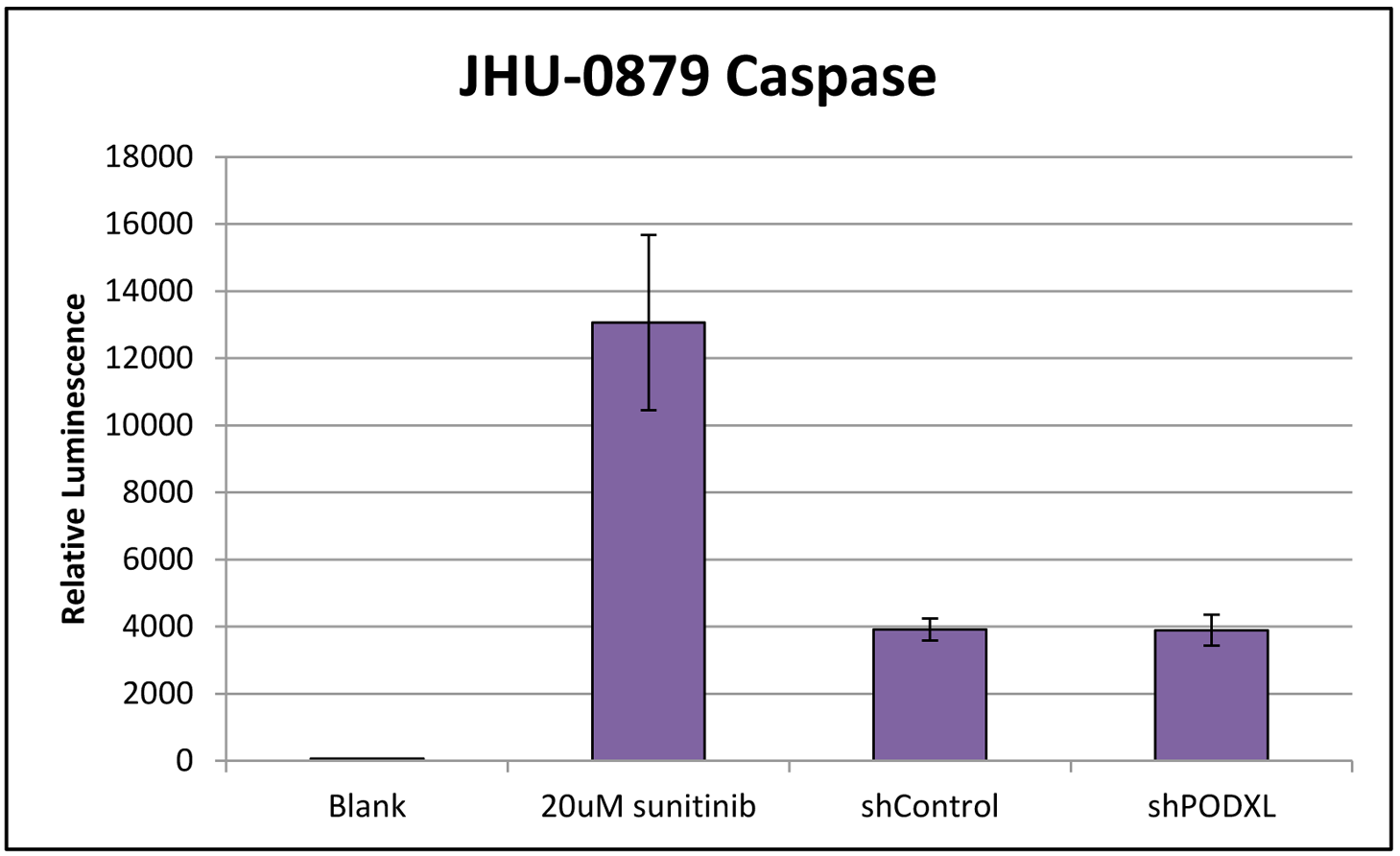

Supplement: Figure S3 — PODXL knockdown does not induce apoptosis. GBM stem-like cell line JHU-0879 was transduced with either shControl or shPODXL and assayed for Caspase 3/7 activity. The positive control was sunitinib at 20 µM and the negative control was wells without cells. There was no apoptosis seen with PODXL knockdown. (TIF) [file pone.0075945.s003.tif]
