# Supplementary material for: Podocalyxin-Like Protein Is Expressed in Glioblastoma Multiforme Stem-Like Cells and Is Associated with Poor Outcome
Source: PLoS One. 2013 Oct 16;8(10):e75945. doi: 10.1371/journal.pone.0075945 (PMC3797817; doi:10.1371/journal.pone.0075945)
Supplement: Table S1 — Genes overexpressed in differentiated GBM oncospheres. (DOCX) [file pone.0075945.s004.docx]

**Table S1.** Genes overexpressed in differentiated GBM oncospheres.

| **SAGE tag sequence** | **Unigene ID** | **Gene symbol (name)** | **Fold incr. 28d** |
| --- | --- | --- | --- |
| GGAAAGTGA | Hs.25647 | FOS (v-fos FBJ murine osteosarcoma viral oncogene homolog) | 34.63 |
| GGATATGTGG | Hs.326035 | EGR1 (early growth response 1) | 16.01 |
| GGGCATCTCT | Hs.520048 | HLA-DRA (major histocompatibility complex, class II, DR alpha) | 15.68 |
| TTGAAAACTC | Hs.437563 | FAM70A (family with sequence similarity 70, member A) | 15.68 |
| CACTTTCTAA | Hs.197043 | MAN1C1 (mannosidase, alpha, class 1C, member 1) | 15.68 |
| GACACGAACA | Hs.25829 | RASD1 (dexamethasone-induced 1 | 14.7 |
| AATGGATTAC | Hs.654638 | LOC497661 (Putative NFkB activating protein) | 13.72 |
| AACACAGCCT | Hs.655564 | C4A (complement component 4A [Rodgers blood group]) | 13.23 |
| AGACAAGTTT | Hs.90093 | HSPA4 (Heat shock 70kDa protein 4) | 11.76 |
| GACCGCGGCT | Hs.505337 | CLDN5 (Claudin 5) | 11.76 |
| GAGAAGCGGC | Hs.154029 | HES4 (Hairy and enhancer of split 4 [Drosophila]) | 10.78 |
| TTTTAGTGTC | Hs.592225 | ARMCX3 (Armadillo repeat containing, X-linked 3) | 10.78 |
| GCCAAGTTCT | Hs.503878 | NCAM1 (Neural cell adhesion molecule 1) | 10.78 |
| CGCTATCTGG | Hs.631988 | DDR1 (Discoidin domain receptor family, member 1) | 9.8 |
| AAACTGTACA | Hs.155017 | NRIP1 (Nuclear receptor interacting protein 1) | 9.8 |
| AAAAATAAAT | Hs.521124 | MRPS28 (Mitochondrial ribosomal protein S28) | 9.8 |
| TTATTCCTCT | Hs.507755 | DCAMKL1 (Doublecortin and CaM kinase-like 1) | 9.8 |
| ATGTATAATA | Hs.499925 | VPS26A (Vacuolar protein sorting 26 homolog A [S. pombe]) | 9.8 |
| TATCCCAGAA | Hs.149360 | ODF2L (outer dense fiber of sperm tails 2-like) | 9.8 |
| TACGGGGATC | Hs.631638 | QTRT1 (Queuine tRNA-ribosyltransferase 1 [tRNA-guanine transglycosylase]) | 9.8 |
| GGGCGAGACC | Hs.533566 | H19 (H19, imprinted maternally expressed untranslated mRNA) | 9.31 |
| GCCAACAACG | Hs.503911 | NNMT (Nicotinamide N-methyltransferase) | 9.31 |
| ACCCACGTCA | Hs.25292 | JUNB (Jun B proto-oncogene) | 9.15 |
| ATGTCACGAC | Hs.349705 | IGFBPL1 (Insulin-like growth factor binding protein-like 1) | 8.82 |
| CCGTAGTGCC | Hs.6891 | SFRS6 (Splicing factor, arginine/serine-rich 6) | 8.82 |
| ATAAAATTCC | Hs.485195 | SORT1 (Sortilin 1) | 8.82 |
| ACCCCCCCGC | Hs.2780 | JUND (Jun D proto-oncogene) | 8.82 |
| AGCTGTTCTG | Hs.546261 | HNRPA1 (Heterogeneous nuclear ribonucleoprotein A1) | 8.82 |
