# Supplementary material for: Podocalyxin-Like Protein Is Expressed in Glioblastoma Multiforme Stem-Like Cells and Is Associated with Poor Outcome
Source: PLoS One. 2013 Oct 16;8(10):e75945. doi: 10.1371/journal.pone.0075945 (PMC3797817; doi:10.1371/journal.pone.0075945)
Supplement: Table S2 — Quantitative real-time PCR expression analysis in three matched sets of undifferentiated and differentiated GBM oncosphere lines. (DOCX) [file pone.0075945.s005.docx]

**Table S2.** Quantitative real-time PCR expression analysis in three matched sets of undifferentiated and differentiated GBM oncosphere lines.

| **Gene** | **020913** | **HSR-GBM2** | **HSR-GBM3** |
| --- | --- | --- | --- |
| **Fold increase in undifferentiated cells** | | | |
| *DUSP6* | 169 | 11 | 4.6 |
| *PODXL* | 21.6 | 6 | 3.8 |
| *PTPRZ1* | 274 | 23.4 | 9 |
| *FABP7* | 52 | 2.6 | - |
| *PDIA4* | 3.3 | 2 | 3.2 |
| **Fold increase in differentiated cells** | | | |
| *FAM70A* | 3.3 | 1.8 | 1.9 |
| *MAN1C1* | 2.2 | 4 | 1.7 |
