# Supplementary material for: Podocalyxin-Like Protein Is Expressed in Glioblastoma Multiforme Stem-Like Cells and Is Associated with Poor Outcome
Source: PLoS One. 2013 Oct 16;8(10):e75945. doi: 10.1371/journal.pone.0075945 (PMC3797817; doi:10.1371/journal.pone.0075945)
Supplement: Table S3 — Pathological diagnoses of the REMBRANDT dataset. (DOCX) [file pone.0075945.s006.docx]

**Table S3.** Pathological diagnoses of the REMBRANDT dataset.

| **Diagnosis** | **Intermediate PODXL Expression** | **Up-regulated PODXL Expression** |
| --- | --- | --- |
| WHO Grade II diffuse and mixed astrocytomas | 47 | 4 |
| WHO Grade II oligodendrogliomas | 19 | 0 |
| WHO Grade III anaplastic oligodendrogliomas | 16 | 5 |
| WHO Grade III anaplastic and mixed anaplastic astrocytomas | 42 | 5 |
| WHO Grade IV GBMs | 111 | 70 |
| Non-specified | 22 | 1 |
